# Synthetic Artificial Apoptosis‐Inducing Receptor for On‐Demand Deactivation of Engineered Cells

**DOI:** 10.1002/advs.202004432

**Published:** 2021-05-01

**Authors:** Pere Monge, Kaja Borup Løvschall, Ane Bretschneider Søgaard, Raoul Walther, Thaddeus W. Golbek, Lars Schmüser, Tobias Weidner, Alexander N. Zelikin

**Affiliations:** ^1^ Department of Chemistry and iNano Interdisciplinary Nanoscience Centre Aarhus University Aarhus 8000 Denmark

**Keywords:** artificial receptors, glucuronide, prodrugs, self‐immolative linkers, signal transduction

## Abstract

The design of a fully synthetic, chemical “apoptosis‐inducing receptor” (AIR) molecule is reported that is anchored into the lipid bilayer of cells, is activated by the incoming biological input, and responds with the release of a secondary messenger—a highly potent toxin for cell killing. The AIR molecule has four elements, namely, an exofacial trigger group, a bilayer anchor, a toxin as a secondary messenger, and a self‐immolative scaffold as a mechanism for signal transduction. Receptor installation into cells is established via a robust protocol with minimal cell handling. The synthetic receptor remains dormant in the engineered cells, but is effectively triggered externally by the addition of an activating biomolecule (enzyme) or in a mixed cell population through interaction with the surrounding cells. In 3D cell culture (spheroids), receptor activation is accessible for at least 5 days, which compares favorably with other state of the art receptor designs.

## Graphical abstract

Artificial receptors afford dedicated communication to the engineered cells. Designed here is a receptor that is anchored into the lipid bilayer, engages with a biological input and affords a biological output, and features a chemical mechanism of signal transduction.

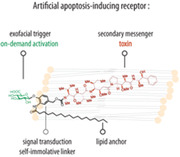

## 1 Introduction

Receptors are an exquisite tool of molecular and cell biology.^[^ ^1^ ^]^ Biological functions of receptors are pivotal for each cell and include recognition of solutes, controlled adhesion events, receptor‐mediated endocytosis, and transmembrane signaling. Design of synthetic artificial receptors is highly challenging but at the same time highly appealing to a broad scientific audience.^[^ ^2^, ^3^, ^4^, ^5^, ^6^, ^7^, ^8^, ^9^, ^10^, ^11^ ^]^ Among the diverse functions of receptors, modulation of adhesion is accomplished well using artificial receptors.^[^ ^6^, ^12^ ^]^ These are created using a successful arsenal of tools of colloidal and surface science, applied to the cell surface. In turn, metabolic engineering has proven to be a highly powerful technique to install artificial chemistry into the cellular membrane, and this has been successful in the context of designing artificial receptors for drug targeting.^[^ ^10^, ^13^, ^14^, ^15^ ^]^ Artificial “receptor‐mediated endocytosis” has also been engineered using bilayer anchored ligands and antibody targeting of synthetic receptors.^[^ ^5^, ^9^ ^]^ Recently, artificial receptors have emerged as a unique orthogonal recognition ligand to engineer highly specific “chimeric antigen receptor T cells” (CAR T)^[^ ^16^, ^17^, ^18^ ^]^ and independently, to boost in vivo CAR T expansion.^[^ ^19^ ^]^ These examples illustrate the highest potential of artificial receptors for the development of cell‐based therapies and broader biomedical engineering. Nevertheless, while cell‐surface sensing and artificial endocytosis are well mimicked by synthetic tools,^[^ ^2^ ^]^ transmembrane signaling using synthetic chemistry remains the highest challenge. Even the most prominent examples of this to date remain simplified compared to the natural counterparts and only perform their function in model lipid bilayers (e.g., liposomes)^[^ ^11^, ^20^ ^]^ whereas their performance within live cells is yet to be achieved.

**Scheme 1:**
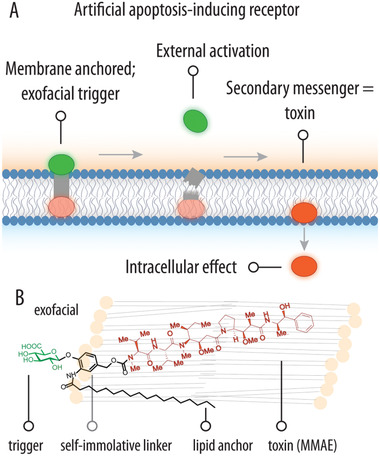
A) Schematic illustration of the proposed artificial apoptosis‐inducing receptor (AIR) design based on a membrane‐bound prodrug with external (exofacial) activation and a secondary messenger molecule; B) chemical formula of the bilayer‐anchored AIR that consists of the glucuronic acid trigger, a C18 lipophilic anchor, *p*‐hydroxybenzyl alcohol self‐immolative linker for drug release, and MMAE as a releasable secondary messenger to exert intracellular response.

Herein, we present the development of a novel approach to externally control the cell fate, using a membrane‐anchored, synthetic, chemical “apoptosis‐inducing receptor” (AIR). Viability switch mechanisms of this kind are highly warranted in the context of cell‐based therapies, such as to establish a possibility to discontinue treatment in an event of severe side effects.^[^ ^21^ ^]^ Apoptosis‐inducing receptors may also prove useful in the context of cancer treatment, where remotely addressable cells act as “Trojan horses” and mediate the killing of surrounding (cancerous) cells.^[^ ^22^, ^23^, ^24^ ^]^ Artificial receptor mechanisms based on biological molecules (chimeric receptors) are highly powerful but are also highly complex in engineering, and for patient derived cells, these require lengthy procedures with extensive cell handling.^[^ ^3^, ^4^, ^19^ ^]^ We hypothesized that the desired artificial receptor can be designed using synthetic chemistry. Thus, we propose an artificial chemical receptor that bears similarity with natural and chimeric counterparts by featuring an exofacial component for receptor activation, a mechanism of signal transduction, and a secondary messenger molecule that exerts intracellular effects (**Scheme**). The AIR molecule designed in this work consists of four fragments: i) an exofacial trigger group that can be cleaved using an externally added enzyme, ii) a membrane anchor, iii) a self‐immolative mechanism for traceless release of the secondary messenger, and iv) a potent liposoluble toxin (Scheme 1B). We demonstrate that receptor installation into cells is a rapid procedure with minimal cell handling, thus making it highly advantageous compared to its chimeric counterparts. Receptor activation can be achieved by externally added biological stimulus (enzyme), or through interaction with surrounding cells.

## 2 Results and Discussion

For receptor activation, we chose to use enzymatic activity and focus on *β*‐glucuronidase (GUS), an enzyme with a unique, predominantly intracellular distribution in the human body.^[^ ^25^, ^26^, ^27^ ^]^ This aspect is pivotal to minimize nonspecific activation of the engineered cells. Indeed, activation of the corresponding substrates, glucuronides, in the human body is only associated with diseased tissues such as cancer, and not with normal healthy tissues.^[^ ^25^, ^26^, ^27^ ^]^ Glucuronides are also highly advantageous for receptor design as being highly polar:^[^ ^28^, ^29^ ^]^ the use of glucuronic acid as a removable masking group is poised to ensure exofacial localization and accessibility of the trigger to enzymatic activation. As an effector molecule, we used a highly potent intracellular toxin, monomethyl auristatin E (MMAE), which has excellent lipophilicity and cell permeability properties (calculated log *P* = 3,5). Finally, the mechanism of triggered drug release is engineered using a self‐immolative *p*‐hydroxylbenzyl alcohol scaffold, which conveniently offers vacant *ortho*‐positions to install a membrane anchoring element, in our case, a C18 aliphatic tail that mimics the lipid bilayer constituents. Self‐immolative linkers (SILs) are highly useful in medicinal chemistry, specifically in the design of prodrugs.^[^ ^29^ ^]^ The innovative aspect of this work lies in fact that we use SIL as a tool for transduction of chemical stimuli across the sealed biomolecular membrane, mimicking performance of natural receptors.

**Figure 1:**
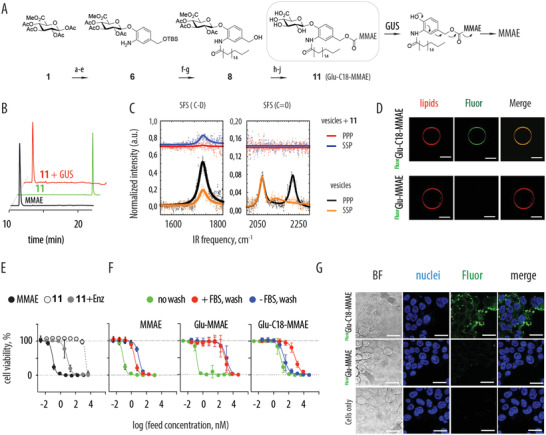
A) Schematic illustration of the chemical route for the synthesis of the AIR molecule (Glu‐C18‐MMAE) and the mechanism for the enzyme‐triggered drug release: B) HPLC characterization of the AIR molecule and the product of enzyme‐triggered drug release (10 × 10^−3^ m PBS, pH 7.4, [GUS] = 0.1 g L^−1^; 2 h at 37 °C); C) sum frequency scattering (SFS) spectroscopy data in the C–D and lipid carbonyl regions in ppp and ssp polarization combinations for deuterated vesicles composed of 1:1 molar ration d62‐DPPC and DPPG lipids in D_2_O. Data illustrate a loss of signal upon addition of Glu‐C18‐MMAE (**11**), indicative of loss of order in the lipid bilayer, due to Glu‐C18‐MMAE anchoring into the lipid bilayer; D) CLSM imaging of rhodamine labeled vesicles upon exposure to Glu‐C18‐MMAE or Glu‐MMAE, the latter two labeled using fluorescein via the Glu‐carboxylic acid functionality. Scale bars: 5 µm. E) Dose response curves for MMAE, Glu‐C18‐MMAE, and Glu‐C18‐MMAE in the presence of GUS enzyme in GUS^Neg^ cells, illustrating masking toxicity of MMAE within the prodrug, which is revealed by added enzyme. F) Representative dose‐response curves for GUS^Neg^ cells incubated with MMAE, Glu‐C18‐MMAE, or Glu‐MMAE and subsequently with GUS enzyme to trigger drug release, with additional 48 h of cell culture before quantification of cell viability. Prodrugs were incubated with cells in the presence or absence of FBS (+FBS/−FBS, respectively), and optionally washed before the addition of GUS enzyme. Presented data are mean ± SD based on at least three independent experiments. For full details, see the experimental conditions. Corresponding IC_50_ values are listed in Table 1. G) CLSM images illustrating GUS^Neg^ cells upon exposure to Fluo‐Glu‐C18‐MMAE or Fluor‐Glu‐MMAE, illustrating successful anchorage of the C18‐containing molecule; scale bars: 6 µm.

The synthesis of the proposed AIR molecule was carried out in 10 steps, starting from protected glucuronic acid **1** (**Figure** **1**A). Glucuronidation of 4‐hydroxy‐3‐nitrobenzaldehyde was conducted under the Königs–Knorr conditions yielding excellent *β*‐selectivity. Subsequently, the aldehyde was reduced into the corresponding benzyl alcohol and the nitro group reduced into an aniline **6**. The aniline functionality ensured the opportunity for the installation of a membrane anchor, which was chosen to be a C18 chain **8**. In turn, benzyl alcohol provided a drug conjugation site, through activation with *p*‐nitrophenyl chloroformate followed by the coupling to the toxin, MMAE. Finally, glucuronic acid was deprotected using Zémplen deacetylation conditions followed by saponification of the methyl ester to afford the desired compound **11** (Glu‐C18‐MMAE). AIR molecule revealed a critical aggregation concentration of 122 × 10^−6^ m; it was stable in solution and exhibited nondetectable spontaneous drug release. Upon addition of GUS, the prodrug underwent a rapid bioconversion and quantitatively released the incorporated drug, MMAE (Figure 1B).

To investigate lipid bilayer anchoring of AIR, we employed sum frequency scattering (SFS) spectroscopy as a tool that can probe molecular vibrations at vesicle surfaces in situ and report back on (bio)molecule‐membrane interactions. The technique employs narrowband visible laser pulses overlapped in space and time with broadband infrared laser pulses; nonlinear optical frequency mixing generates sum‐frequency photons, which carry a vibrational spectrum of the particle surface and provide information about the order and alignment of interfacial species.^[^ ^30^, ^31^, ^32^ ^]^ For neat vesicles prepared using deuterated lipids, the C=O stretching region of the spectrum (the lipid head groups at the vesicle interface) and the C–D region of the spectrum (resonances near 2075, 2125, and 2225 cm^−1^ assigned to the symmetric CD_3_, CD_3_ Fermi resonance, and symmetric CD_3_, respectively) reveal resonances related to ordered lipid molecules. The intensity of these modes was drastically reduced upon exposure to Glu‐C18‐MMAE (Figure 1C), whereas dynamic light scattering profiles of the vesicles remained unchanged (Figure S3, Supporting Information). This data illustrates a severe reduction of lipid order within otherwise intact vesicles in the presence of Glu‐C18‐MMAE, which is indicative of AIR inserting into lipid bilayer membrane.

Next, we labeled AIR molecule using the glucuronic acid carboxyl group, using an amine‐containing derivative of fluorescein (for synthesis and characterization, see the Supporting Information). Fluorescein can also be used as an exofacial ligand,^[^ ^33^ ^]^ which is important to maintain the overall amphiphilic character of the AIR molecule. Confocal laser scanning microscopy (CLSM) reveals that upon administration onto giant unilamellar vesicles (GUVs), the AIR molecule spontaneously incorporates into the lipid bilayer, as evidenced by strong fluorescent signal corresponding to fluorescein (Figure 1D). This behaviour was not observed for glucuronide of MMAE that is devoid of the C18‐lipid anchor (Glu‐MMAE). Together, SFS spectroscopy and CLSM validate spontaneous lipid bilayer insertion for the AIR molecule, as is pivotal for its application as an artificial receptor.

For cell culture evaluation, we used a GUS‐knock out derivative of the HAP‐1 human myeloid leukemia‐derived cells (GUS^Neg^) as well as its parent, GUS‐competent counterpart. Dose response curves were obtained for the synthesized AIR molecule (Glu‐C18‐MMAE) and its simplified analogue devoid of the C18 anchor, Glu‐MMAE. Representative dose‐response curves are shown in Figure 1E,F; IC_50_ values derived thereof are listed in **Table** **1**.

**Table 1:** IC_50_ values of Glu‐C18‐MMAE, Glu‐MMAE, and MMAE in GUS^Neg^ cells and GUS‐competent HAP1 cells under different washing procedures, with and without enzyme treatment. QIC_50_ is defined as fold‐difference between IC_50_ values observed for the prodrug in the absence or in the presence of externally added enzyme. All experiments were reproduced three times with three replicates each time (*N* = 3, *n* = 3)

IC_50_ [nm]							
Condition		GUS^Neg^ cells			GUS competent cells		
		−enz	+enz	QIC_50_	−enz	+enz	QIC_50_
No wash	MMAE	–	0.10 ± 0.03	–	–	0.10 ± 0.04	–
	Glu‐MMAE	300 ± 15	0.20 ± 0.08	1497	89 ± 23	0.20 ± 0.04	465
	Glu‐C18‐MMAE	2700 ± 600	7.0 ± 1.2	397	330 ± 20	8.8 ± 1.0	37
Wash	MMAE	–	6.9 ± 0.7	–	–	8.7 ± 0.5	–
	Glu‐MMAE	5100 ± 700	1300^ ^± 270	4	3700 ± 500	1200 ± 200	3
	Glu‐C18‐MMAE	>10^4^	730 ± 70	25	940 ± 60	450 ± 30	2
Serum‐free	MMAE	–	2.7 ± 0.2	–	–	2.8 ± 0.2	–
	Glu‐MMAE	>10^4^	850 ± 140	4	>10^4^	730 ± 160	17
	Glu‐C18‐MMAE	3100 ± 1200	24 ± 3	132	59 ± 6	14 ± 2	4

Designed compounds act as prodrugs for MMAE: sub‐nanomolar toxicity of MMAE is masked to a micromolar level by the glucuronides, and is restored to the nanomolar range in the presence of GUS enzyme (Figure 1E and Table 1), as is well documented.^[^ ^28^, ^34^ ^]^ These results demonstrate that glucuronides are highly effective as prodrugs and fold‐change in toxicity‐related IC_50_ values (defined as QIC_50_) exceeds 100, which provides a highly favorable safety window. However, the synthesized glucuronides, regardless of the presence of the C18 anchor, exhibited only minor if any anchoring into the cell membranes if administered onto cells in the presence of serum. Indeed, a simple washing step implemented after a 2 h (pro)drug incubation with cells, but prior to addition of the activating enzyme, removes 99+% of the (pro)drug, as evidenced by an ≈100–1000‐fold increase in the apparent IC_50_ values (Figure 1F, c. f. “no wash” and “+FBS, wash”). This is observed for each of the three solutes, including pristine MMAE and Glu‐C18‐MMAE. In other words, at these conditions, glucuronides act as prodrugs and not as membrane‐bound receptors.

Cell membrane anchoring for the Glu‐C18‐MMAE was successfully achieved through administration and brief incubation of cells with AIR in serum‐free media, that is, conditions that are routinely employed for cell isolation and sorting during, e.g., engineering of CAR T. Subsequent cell culture was conducted in full serum, that is, standard cell culture conditions. CLSM reveals strong fluorescence of cells upon exposure to the fluorescently labeled derivative of AIR, which is not observed for pristine cells or the fluorescently labeled glucuronide Glu‐MMAE (Figure 1G). Cell culture evaluation revealed that with this mode of administration, IC_50_ values for toxicity were 2.7 × 10^−9^ m for MMAE and micromolar for Glu‐MMAE prodrug (no bilayer anchoring). IC_50_ value for the Glu‐C18‐MMAE was 23 × 10^−9^ m, which is only tenfold higher than for MMAE at these cell culture conditions. The IC_50_ value is also only threefold higher than the value observed through activation of the total administered prodrug payload in solution (the “no wash” conditions), which suggests that ≈30% of the administered prodrug was anchored into the lipid bilayer.

Most importantly, the anchored Glu‐C18‐MMAE remains dormant in GUS^Neg^ cells. In the absence of the enzyme, spontaneous drug release affords an IC_50_ value of over 3 × 10^−6^ m, which is ≈130‐fold higher than in the presence of the enzyme (24 × 10^−9^ vs 3100 × 10^−9^ m in GUS^Neg^ cells, in the presence and absence of added enzyme, respectively). This offers a highly favorable safety window to achieve remotely triggered deactivation of cells that are equipped with the dormant AIR receptor molecule using externally added enzyme. From a different perspective, under the same anchorage conditions, Glu‐C18‐MMAE in GUS‐competent cells, with no external addition of GUS enzyme, exhibited an IC_50_ value of ≈59 × 10^−9^ m. This illustrates a 50‐fold selectivity window for differential toxicity in GUS^Neg^ and GUS+ cells (3100 × 10^−9^ vs 59 × 10^−9^ m in GUS^Neg^ vs GUS‐competent cell, respectively), for potential cell‐based drug delivery applications.

**Figure 2:**
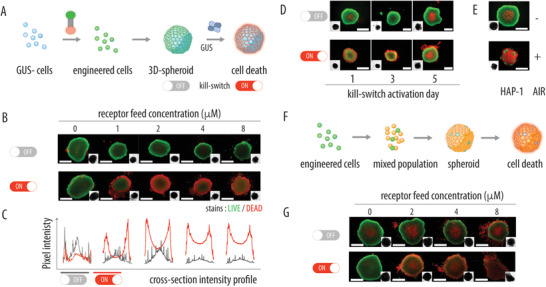
A) Schematic illustration of the 3D cell culture approach that consists of engineering cells using the AIR molecule, cell organization into spheroids, and subsequent cell culture and receptor activation. B) Fluorescence microscopy images for GUS^Neg^ cell spheroids engineered using apoptosis‐inducing receptor at varied receptor feed concentrations, with or without receptor activation; C) cross‐section intensity profile analyses for the fluorescence images shown in panel B; D) fluorescence microscopy images illustrating viability of the AIR‐containing GUS^Neg^ cells within spheroids with receptor activation at day 1, day 3, and day 5 (receptor feed concentration during cell engineering 1 × 10^−6^ m); E) Live (green)/Dead (red) fluorescence microscopy images of 3 day old spheroids grown using GUS‐competent HAP‐1 cells with or without AIR molecule: receptor feed concentration 1 μM; no external enzyme added for receptor activation; scale bars 500 μm; F) schematic illustration of assembly for the mixed cell spheroids; G) fluorescence microscopy images illustrating cell viability within mixed cell spheroids as a function of receptor feed concentration during cell engineering, for a “Trojan horse”‐type receptor activation (trigger “off”) and receptor activation using externally added GUS enzyme (trigger “on”). For full details, see Supporting Information.

Taken together, results of cell culture evaluation for the synthesized AIR molecule indicate that in GUS^Neg^ cells, AIR is dormant in a wide range of concentrations and can be activated to release the incorporated toxin, with a 130‐fold therapeutic index over nontriggered drug release and a 50‐fold therapeutic index over the GUS‐competent cells. To capitalize on these opportunities, we investigated triggered deactivation of engineered cells in 3D cells culture, using cell spheroids (**Figure** **2**A). GUS^Neg^ cells engineered to contain AIR formed well‐defined spheroids that could be grown for extended times (at least 7 days). At AIR feed content up to 8 × 10^−6^ m, we observed no spontaneous toxicity to cells, as evidenced by fluorescence microscopy observation (Figure 2B) and fluorescence quantification (Figure 2C). Dormant AIR could be activated by addition of GUS enzyme, which resulted in pronounced toxicity to cells, which was observed with receptor feed concentration as low as 1 × 10^−6^ m. This illustrates successful engineering of mammalian cells to contain a dormant, artificial apoptosis‐inducing receptor that can be activated to achieve an on‐demand cell killing.

Dormant AIR could be activated on demand for at least 5 days of spheroid incubation (Figure 2D). Over this time, AIR is possibly diluted through cell division, through redistribution into solution phase (due to association with serum proteins), and redistribution into intracellular compartments. Prior reports on cell surface engineering (design of artificial glycocalyx) revealed a fast loss of exofacial membrane‐bound ligands, with kinetics of loss measured in hours.^[^ ^6^ ^]^ Thus, the synthetic receptor molecule engineered in this work exhibits membrane persistence well exceeding that of the lipids, which is highly advantageous. The most membrane‐persistent anchor reported previously is cholesterylamine, for which exofacial presence is due to continuous recycling from cell surface into the intracellular compartments and back to the cell surface, over multiple days.^[^ ^5^, ^6^ ^]^ Yet even in this case, artificial glycocalyx has been shown to perform the nominated function over a time frame of 27 h (in vivo, in zebrafish).^[^ ^6^ ^]^ Compared to this, 5 days of accessibility of AIR in 3D cell culture presents a considerable advance. In part, this is due to the design of the receptor, based on the C18 aliphatic lipid bilayer anchor and MMAE, which is a lipophilic toxin and contributes to membrane anchoring. However, to a greater extent, we believe this is due to the chosen 3D (spheroid) cell culture format, while in classical 2D cell culture, we observed a significantly shorter duration of accessibility of the receptor (not exceeding 2 days). Finally, we note that it may be possible to extend the on‐demand activation opportunities beyond 5 days through the use of cholesterolamine, for receptor recycling via natural cell biology mechanisms; this is the subject of ongoing research.

From a different perspective, we also considered that engineered cells can act as “Trojan horses” for delivery of the dormant AIR molecule to diseased tissues (e.g., for cancer treatment). Indeed, white blood cells such as monocytes and lymphocytes are known to infiltrate into tissues and specifically into the disease‐affected areas. Immune cells gain access through the most tightly guarded barriers such as the blood brain barrier.^[^ ^24^ ^]^ Cell‐based therapies are gaining momentum and engineered cells equipped with AIR may be envisioned as efficient cell‐based drug carriers.^[^ ^24^, ^35^ ^]^ We considered that engineered cells can exert toxicity with at least three alternative mechanisms. Firstly, toxicity exerted by the engineered cells can be due to receptor “sharing,” from the infiltrating engineered cells to the surrounding cells comprising the target tissue. Whereby infiltrating donor cells are GUS^Neg^, the acceptor cells are GUS‐competent and therefore will be capable of activating AIR (Figure 2E, also c.f. IC_50_ values, Table 1). Second, cancerous and inflamed tissues are characterized by the presence of extracellular GUS.^[^ ^25^, ^26^, ^27^ ^]^ Elsewhere in the body GUS is confined to the intracellular compartments, and extracellular GUS can be used as a marker for detection and quantification of cancer tissue growth.^[^ ^25^ ^]^ Extracellular GUS can therefore activate artificial receptor designed in this work, in a manner much similar to that illustrated in Figure 1. Finally, receptor activation can be achieved remotely, via external addition of GUS enzyme. Worthy of note, receptor design featuring MMAE is beneficial in that this toxin is characterized by a strongly pronounced bystander effect (due to ease of membrane permeability of MMAE).^[^ ^36^ ^]^ Indeed, we observed successful cell killing in 3D cell spheroids assembled using mixed population of cells (GUS^Neg^ and GUS‐competent). “Trojan horse” toxicity was achieved within spheroids that contained 10% engineered cells without external addition of GUS enzyme (Figure 2F,G). For this, engineering of the donor cells was performed using 8 × 10^−6^ m feed concentration of the receptor. At this concentration, spheroids assembled only GUS^Neg^ cells showed no signs of cell death (Figure 2B). In contrast, in a mixed cell spheroid, with no externally added enzyme, we observed significant cell killing (Figure 2G). It was further possible to trigger the AIR using externally added enzyme. On‐demand toxicity to the mixed cell spheroid was achieved using GUS^Neg^ cells engineered using as low as 2 × 10^−6^ m feed concentration of the receptor (Figure 2G).

## 3 Conclusions

This study presents the design of a molecular switch, a synthetic apoptosis‐inducing receptor, for an on‐demand deactivation of cells. This receptor contains i) an exofacial trigger for enzymatic activation of drug release, ii) a highly potent liposoluble toxin, MMAE, iii) an additional membrane anchor (C18 aliphatic side group), and iv) a self‐immolative scaffold as a signal transduction mechanism. Compared to chimeric (protein‐based) artificial receptors, synthetic chemical receptor designed in this work is installed into mammalian cells within minutes, with minimal cell handling, which is highly appealing for practical applications of engineered cells. We demonstrated on‐demand deactivation of cells in 2D and in 3D cell culture, in monoculture and in mixed cell coculture. We believe our data open up diverse opportunities for the use of engineered cells in biotechnology and biomedicine, specifically communication to the engineered cells via a dedicated route and cell‐based drug delivery.

## Conflict of Interest

The authors declare no conflict of interest.

## Supporting information

Supporting Information

## Data Availability

Research data are not shared.

## References

[1] R. Santos , O. Ursu , A. Gaulton , A. P. Bento , R. S. Donadi , C. G. Bologa , A. Karlsson , B. Al‐Lazikani , A. Hersey , T. I. Oprea , J. P. Overington , Nat. Rev. Drug Discovery 2017, 16, 19. 27910877 10.1038/nrd.2016.230 PMC6314433

[2] P. Monge , A. B. Søgaard , D. G. Andersen , R. Chandrawati , A. N. Zelikin , Adv. Drug Delivery Rev. 2021, 170, 281. 10.1016/j.addr.2021.01.010 33486005

[3] L. Morsut , K. T. Roybal , X. Xiong , R. M. Gordley , S. M. Coyle , M. Thomson , W. A. Lim , Cell 2016, 164, 780. 26830878 10.1016/j.cell.2016.01.012 PMC4752866

[4] K. T. Roybal , L. J. Rupp , L. Morsut , W. J. Walker , K. A. McNally , J. S. Park , W. A. Lim , Cell 2016, 164, 770. 26830879 10.1016/j.cell.2016.01.011 PMC4752902

[5] D. Hymel , B. R. Peterson , Adv. Drug Delivery Rev. 2012, 64, 797. 10.1016/j.addr.2012.02.007 PMC3359398 22401875

[6] E. C. Woods , N. A. Yee , J. Shen , C. R. Bertozzi , Angew. Chem., Int. Ed. Engl. 2015, 54, 15782. 26647316 10.1002/anie.201508783 PMC4736730

[7] H. P. Dijkstra , J. J. Hutchinson , C. A. Hunter , H. Qin , S. Tomas , S. J. Webb , N. H. Williams , Chem. ‐ Eur. J. 2007, 13, 7215. 17576643 10.1002/chem.200601723

[8] T. Schrader , M. Maue , M. Ellermann , J. Recept. Signal Transduction 2006, 26, 473. 10.1080/10799890600950545 17118794

[9] S. Boonyarattanakalin , S. E. Martin , S. A. Dykstra , B. R. Peterson , J. Am. Chem. Soc. 2004, 126, 16379. 15600339 10.1021/ja046663o

[10] H. Wang , R. Wang , K. Cai , H. He , Y. Liu , J. Yen , Z. Wang , M. Xu , Y. Sun , X. Zhou , Q. Yin , L. Tang , I. T. Dobrucki , L. W. Dobrucki , E. J. Chaney , S. A. Boppart , T. M. Fan , S. Lezmi , X. Chen , L. Yin , J. Cheng , Nat. Chem. Biol. 2017, 13, 415. 28192414 10.1038/nchembio.2297 PMC5458775

[11] M. J. Langton , F. Keymeulen , M. Ciaccia , N. H. Williams , C. A. Hunter , Nat. Chem. 2017, 9, 426. 28430205 10.1038/nchem.2678

[12] N. Lahav‐Mankovski , P. K. Prasad , N. Oppenheimer‐Low , G. Raviv , T. Dadosh , T. Unger , T. M. Salame , L. Motiei , D. Margulies , Nat. Commun. 2020, 11, 1299. 32157077 10.1038/s41467-020-14336-7 PMC7064574

[13] S. Lee , H. Koo , J. H. Na , S. J. Han , H. S. Min , S. J. Lee , S. H. Kim , S. H. Yun , S. Y. Jeong , I. C. Kwon , K. Choi , K. Kim , ACS Nano 2014, 8, 2048. 24499346 10.1021/nn406584y

[14] H. Wang , M. C. Sobral , T. Snyder , Y. Brudno , V. S. Gorantla , D. J. Mooney , Biomater. Sci. 2020, 8, 266. 31690897 10.1039/c9bm01487j

[15] A. Uvyn , R. De Coen , O. De Wever , K. Deswarte , B. N. Lambrecht , B. G. De Geest , Chem. Commun. 2019, 55, 10952. 10.1039/c9cc03379c 31441915

[16] Y. G. Lee , I. Marks , M. Srinivasarao , A. K. Kanduluru , S. M. Mahalingam , X. Liu , H. Chu , P. S. Low , Cancer Res. 2019, 79, 387. 30482775 10.1158/0008-5472.CAN-18-1834

[17] J. S. Ma , J. Y. Kim , S. A. Kazane , S. H. Choi , H. Y. Yun , M. S. Kim , D. T. Rodgers , H. M. Pugh , O. Singer , S. B. Sun , B. R. Fonslow , J. N. Kochenderfer , T. M. Wright , P. G. Schultz , T. S. Young , C. H. Kim , Y. Cao , Proc. Natl. Acad. Sci. USA 2016, 113, E450. 26759368 10.1073/pnas.1524193113 PMC4743826

[18] Y. Cao , D. T. Rodgers , J. Du , I. Ahmad , E. N. Hampton , J. S. Ma , M. Mazagova , S. H. Choi , H. Y. Yun , H. Xiao , P. Yang , X. Luo , R. K. Lim , H. M. Pugh , F. Wang , S. A. Kazane , T. M. Wright , C. H. Kim , P. G. Schultz , T. S. Young , Angew. Chem., Int. Ed. Engl. 2016, 55, 7520. 27145250 10.1002/anie.201601902 PMC5207029

[19] L. Ma , T. Dichwalkar , J. Y. H. Chang , B. Cossette , D. Garafola , A. Q. Zhang , M. Fichter , C. Wang , S. Liang , M. Silva , S. Kumari , N. K. Mehta , W. Abraham , N. Thai , N. Li , K. D. Wittrup , D. J. Irvine , Science 2019, 365, 162. 31296767 10.1126/science.aav8692 PMC6800571

[20] F. G. A. Lister , B. A. F. Le Bailly , S. J. Webb , J. Clayden , Nat. Chem. 2017, 9, 420.

[21] C. E. Brown , C. L. Mackall , Nat. Rev. Immunol. 2019, 19, 73. 30631206 10.1038/s41577-018-0119-y

[22] M. R. Choi , K. J. Stanton‐Maxey , J. K. Stanley , C. S. Levin , R. Bardhan , D. Akin , S. Badve , J. Sturgis , J. P. Robinson , R. Bashir , N. J. Halas , S. E. Clare , Nano Lett. 2007, 7, 3759. 17979310 10.1021/nl072209h

[23] A. T. Power , J. C. Bell , Gene Ther. 2008, 15, 772. 18369325 10.1038/gt.2008.40

[24] E. V. Batrakova , H. E. Gendelman , A. V. Kabanov , Expert Opin. Drug Delivery 2011, 8, 415. 10.1517/17425247.2011.559457 PMC3062753 21348773

[25] J. Lange , B. Eddhif , M. Tarighi , T. Garandeau , E. Péraudeau , J. Clarhaut , B. Renoux , S. Papot , P. Poinot , Angew. Chem., Int. Ed. Engl. 2019, 58, 17563. 31518472 10.1002/anie.201906261

[26] I. F. Antunes , H. J. Haisma , P. H. Elsinga , A. van Waarde , A. T. Willemsen , R. A. Dierckx , E. F. de Vries , Mol. Imaging 2012, 11, 7290.2011.00029. 22418030

[27] A. N. Zelikin , Nat. Chem. 2020, 12, 11.31836871

[28] M. T. Jarlstad Olesen , R. Walther , P. P. Poier , F. Dagnæs‐Hansen , A. N. Zelikin , Angew. Chem., Int. Ed. 2020, 59, 7390. 10.1002/anie.201916124 32073708

[29] R. Walther , J. Rautio , A. N. Zelikin , Adv. Drug Delivery Rev. 2017, 118, 65. 10.1016/j.addr.2017.06.013 28676386

[30] A. G. F. de Beer , S. Roke , Phys. Rev. B 2009, 79, 155420.

[31] S. Roke , W. G. Roeterdink , J. E. G. J. Wijnhoven , A. V. Petukhov , A. W. Kleyn , M. Bonn , Phys. Rev. Lett. 2003, 91, 258302. 14754165 10.1103/PhysRevLett.91.258302

[32] J. Franz , T. Bereau , S. Pannwitt , V. Anbazhagan , A. Lehr , U. Nubbemeyer , U. Dietz , M. Bonn , T. Weidner , D. Schneider , Chem. ‐ Eur. J. 2017, 23, 9690. 28504864 10.1002/chem.201702041

[33] P. Monge , A. Tvilum , A. B. Søgaard , K. B. Løvschall , M. T. Jarlstad Olesen , A. N. Zelikin , Adv. Sci. 2020, 7, 2001395. 10.1002/advs.202001395 PMC7509642 32999846

[34] R. Walther , M. T. Jarlstad Olesen , A. N. Zelikin , Org. Biomol. Chem. 2019, 17, 6970. 31290904 10.1039/c9ob01384a

[35] E. Bender , Nature 2016, 540, S106. 28002399 10.1038/540S106a

[36] N. Joubert , C. Denevault‐Sabourin , F. Bryden , M. C. Viaud‐Massuard , Eur. J. Med. Chem. 2017, 142, 393. 28911823 10.1016/j.ejmech.2017.08.049

