# Supplementary material for: Synthetic Artificial Apoptosis‐Inducing Receptor for On‐Demand Deactivation of Engineered Cells
Source: Adv Sci (Weinh). 2021 May 1;8(13):2004432. doi: 10.1002/advs.202004432 (PMC9539725; doi:10.1002/advs.202004432)
Supplement: Supplementary file 1 — Supporting Information [file ADVS-8-2004432-s001.pdf]

© 2021 Wiley-VCH GmbH

ADVANCED SCIENCE
Open Access

# Supporting Information

for *Adv. Sci.*, DOI: 10.1002/advs.202004432

# Synthetic artificial apoptosis-inducing receptor for on-demand deactivation of engineered cells

*Pere Monge, Kaja Borup Løvschall, Ane Bretschneider Søgaard, Raoul Walther, Thaddeus W. Golbek, Lars Schmüser, Tobias Weidner, and Alexander N. Zelikin**

# Synthetic artificial apoptosis-inducing receptor for on-demand deactivation of engineered cells

Pere Monge,[#] Kaja Borup Løvschall,[#] Ane Bretschneider Søgaard, Raoul Walther, Thaddeus W. Golbek, Lars Schmüser, Tobias Weidner, and Alexander N. Zelikin[*]

Department of Chemistry and iNano Interdisciplinary Nanoscience Centre
Aarhus University, Aarhus 8000 Denmark
Email : zelikin@chem.au.dk

## Supporting information

# Contents

1. General information ... 3
2. Chemistry section ... 3
2.1 Synthesis overview ... 3
2.2 Synthetic protocols ... 4
2.2.1 Synthesis of Compound 2 ... 4
2.2.2 Synthesis of Compound 3 ... 4
2.2.3 Synthesis of Compound 4 ... 5
2.2.4 Synthesis of Compound 5 ... 5
2.2.5 Synthesis of Compound 6 ... 6
2.2.6 Synthesis of Compound 7 ... 6
2.2.7 Synthesis of Compound 8 ... 7
2.2.8 Synthesis of Compound 9 ... 8
2.2.9 Synthesis of Compound 10 ... 8
2.2.10 Synthesis of Compound 11 ... 9
2.2.11 Synthesis of FITC-cadaverine (Compound 12) ... 9
2.2.12 Fluorescent labelling of Glu-C18-MMAE and Glu-MMAE (Compounds 13 and 14). ... 10
2.3 NMR spectra ... 13
2.4 MS spectra ... 21
2.5 HPLC drug release experiment ... 24
3. Physics section ... 24
3.1 Phospholipid Vesicle and Sample Preparation ... 24
3.2 Dynamic light scattering (DLS) ... 24
3.3 Sum frequency scattering (SFS) spectroscopy ... 25
3.4 Critical micellar concentration (CMC) of Glu-C18-MMAE ... 27
3.5 GUVs formation and binding studies with fluorescently labelled compounds ... 27
4. Biology section ... 28
4.1 Cell culture ... 28
4.2 HAP1 and bGUS knockout cell lines ... 28
4.3 Spheroid formation and culture ... 28
4.4 Determination of IC50 ... 28
4.4 Live/Dead staining spheroids ... 28

4.5 Activation of synthetic “kill switch” in 3D cultures of bGUS knockout cells .......................... 28
4.6 Co-culture experiments in 3D cell cultures .......................... 29
4.7 Confocal laser scanning microscopy of fluorescently labelled compounds on cell culture ..... 29
4.8 Dose response curves .......................... 30
4.9 Statistical analysis .......................... 31
5. References .......................... 32

# 1. General information

Unless stated otherwise, all chemicals were purchased from Sigma Aldrich and used without further purification. Triethylamine, *N,N*-dimethylformamide, and methanol were obtained anhydrous. Monomethyl auristatin E (MMAE) was acquired from ApiChem. The deuterated solvents used for NMR were purchased from EurisoTop. The solvents dichloromethane, acetonitrile, and tetrahydrofurane were collected from a MBraun SP800 purification system. Glu-MMAE was synthesized according to a protocol developed previously in the group.[1]

Nuclear magnetic resonance (NMR) spectra were recorded from a Bruker BioSpin Gmbh 400 MHz spectrometer or a Varian Mercury 400 MHz spectrometer. The spectra were taken either as $^{1}$H-NMR 400 MHz or $^{13}$C-NMR 101 MHz and they were referenced to the solvent peak. High-resolution mass spectrometry (HR-MS) was carried out with a Bruker Maxis Impact-TOF-MS with electrospray ionization (ESI). The high-performance liquid chromatography (HPLC) experiments were performed on a Shimadzu LC2010-HT equipped with a C18 Express-peptide column from Ascentis with following specifications; a particle size of 2.7 µm, a length of 150 mm and a diameter of 3.0 mm). The mobile phases consisted of ultrapure water with trifluoroacetic acid (TFA) (0.1% v/v%) (eluent A) and HPLC grade acetonitrile with TFA (0.1% v/v%) (eluent B).

# 2. Chemistry section

## 2.1 Synthesis overview

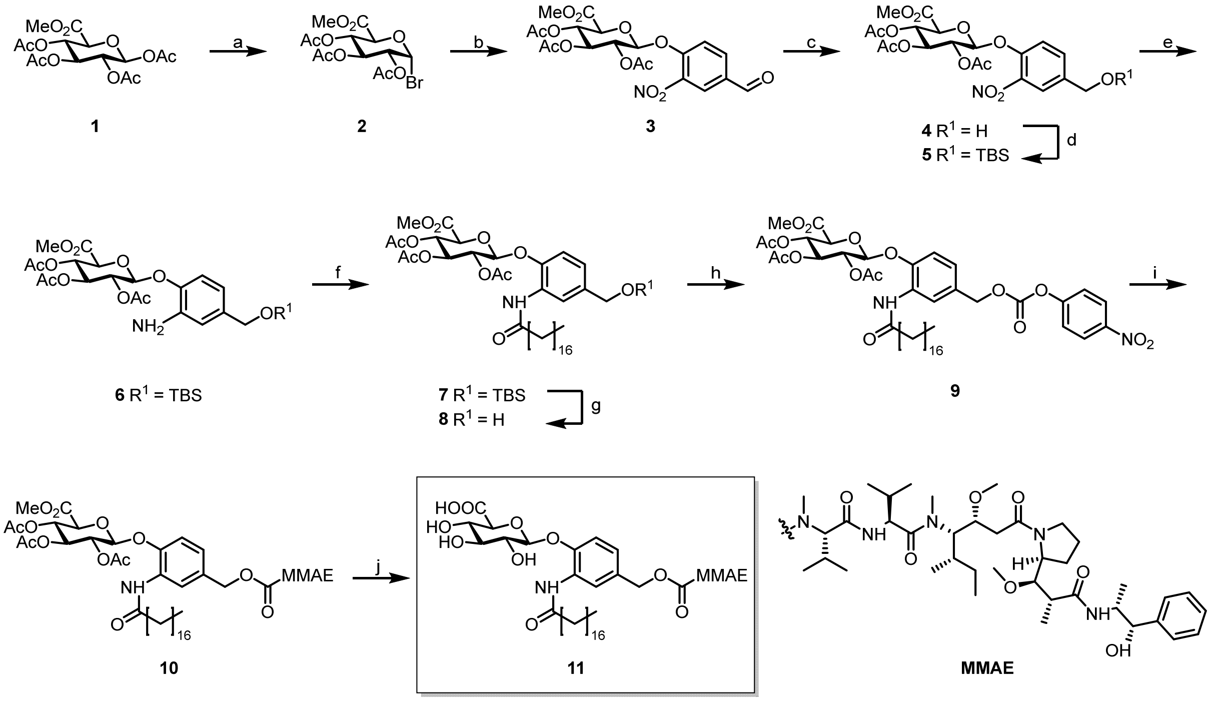


**Figure S1**: Complete synthesis of compound **11**. Conditions and reagents; a) HBr/AcOH, $CH_2Cl_2$, 0ºC to r.t., 4 h, 69%, b) 4-hydroxy-3-nitrobenzaldehyde, powdered 3Å molecular sieves, $Ag_2O$, $CH_3CN$, r.t., 18 h, 84%, c) $NaBH_4$, silica gel, isopropanol/$CHCl_3$, 0ºC, 1.5 h, 80%, d) TBSCl, imidazole, DMAP, DMF, r.t, 42 h, 86%, e) Pd/C, ammonium formate, abs. EtOH, r.t., 3 h, 95%, f) steraoyl chloride, TEA, $CH_2Cl_2$, 0ºC to r.t., 4 h, 87%, g) TEA·3HF, THF, 0ºC to r.t., 40.5 h, 74%, h) 4-nitrophenyl chloroformate, TEA, $CH_2Cl_2$, 0ºC to r.t., 21 h, 68%, i) MMAE, HOBt, TEA, DMF, 0ºC to r.t., 46 h, 88%, j) NaOMe, NaOH, MeOH, 0ºC to r.t., 25%.

## 2.2 Synthetic protocols

### 2.2.1 Synthesis of Compound 2

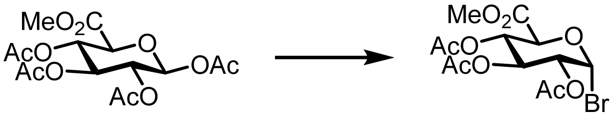

**1** **2**

Compound **2** was obtained through a similar protocol as described in literature[2]. Compound **1** (1.98 g, 1 equiv.) and $CH_2Cl_2$ (10 mL) were mixed in a flame-dried flask under argon atmosphere and the solution was cooled to 0°C. HBr/AcOH (20 mL) was slowly added and the resulting mixture was left stirring at room temperature for four hours. The crude reaction mixture was poured onto ice, the organic phase was washed with ice water (3x30 mL) and saturated $NaHCO_3$ (3x30 mL), dried over $MgSO_4$, and filtered. The filtrate was collected and the solvent was removed under reduced pressure to yield the desired Compound **2** as a brown syrup (1.46 g, 69%). **$^1$H-NMR** (400 MHz, Chloroform-*d*) δ (ppm) 6.64 (d, $J$ = 4.1 Hz, 1H), 5.61 (t, $J$ = 9.7 Hz, 1H), 5.24 (dd, $J$ = 10.3, 9.5 Hz, 1H), 4.85 (dd, $J$ = 10.0, 4.1 Hz, 1H), 4.58 (dd, $J$ = 10.3, 0.7 Hz, 1H), 3.76 (s, 3H), 2.10 (s, 3H), 2.06 (s, 3H), 2.05 (s, 3H). **$^{13}$C-NMR** (101 MHz, Chloroform-*d*) δ (ppm) 169.7, 169.7, 169.5, 166.7, 85.3, 72.0, 70.3, 69.3, 68.5, 53.2, 20.6 (2C), 20.5. **HR-MS** (ESI): calcd. for $[C_{13}H_{17}BrO_9 + H]^+$: 397.0129, found 397.0711.

### 2.2.2 Synthesis of Compound 3

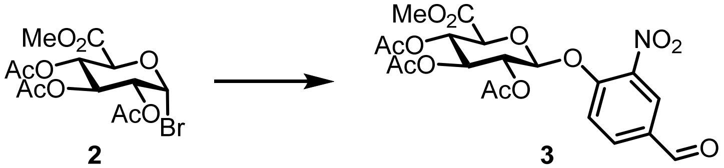

**2** **3**

Compound **3** was prepared similar to a protocol described in literature[2]. Compound **2** (1.46 g, 1 equiv.), 4-hydroxy-3-nitrobenzaldehyde (1.23, 2 equiv.), and powdered 3Å molecular sieves (1.5 g) were added under an atmosphere of argon to a flame-dried Schlenk flask. Furthermore, $CH_3CN$ (45 mL) was added to the flask and the suspension was left stirring at room temperature. After 30 minutes, $Ag_2O$ (1.71 g, 2 equiv.) was added and the mixture was stirred for 18 hours in the dark at room temperature. Upon completion, the reaction mixture was filtered over a plug of Celite® and washed with $CH_2Cl_2$. The resulting filtrate was concentrated *in vacuo* and the crude was purified by silica column chromatography eluting with EtOAc:pentane 1:3 to 3:2 yielding Compound **3** (1.50 g, 84%). **$^1$H-NMR** (400 MHz, Acetone-*d*$_6$) δ (ppm) 10.05 (s, 1H), 8.40 (d, $J$ = 2.0 Hz, 1H), 8.22 (dd, $J$ = 8.7, 2.1 Hz, 1H), 7.78 (d, $J$ = 8.7 Hz, 1H), 5.88 (d, $J$ = 7.4 Hz, 1H), 5.47 (t, $J$ = 9.2 Hz, 1H), 5.36 – 5.22 (m, 2H), 4.73 (d, $J$ = 9.5 Hz, 1H), 3.68 (s, 3H), 2.04 (s, 3H), 2.02 (s, 3H), 2.00 (s, 3H). **$^{13}$C-NMR** (101 MHz, Acetone-*d*$_6$) δ (ppm) 190.4, 170.2, 170.0, 169.5, 167.7, 153.7, 135.2, 132.6, 126.9, 119.1, 99.2, 73.0, 71.7, 70.9, 69.7, 53.1, 20.6, 20.5, 20.5. **HR-MS** (ESI): calcd. for $[C_{20}H_{21}NO_{13}+Na]^+$: 506.0905, found 506.0917, calcd. for $[C_{20}H_{21}NO_{13}+K]^+$: 522.0645, found 522.0646.

### 2.2.3 Synthesis of Compound 4

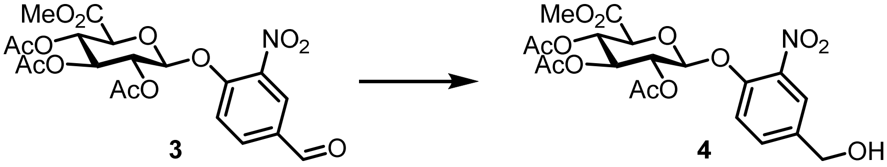


Compound **4** was synthesized by a similar protocol as described in literature[2]. Compound **3** (1.42 g, 1 equiv.) was added to a flame-dried flask and dissolved in a mixture of $CHCl_3$/isopropanol (25.0 mL/6 mL) under an atmosphere of argon. The solution was cooled to 0ºC and silica gel (1.3 g) was further added. The mixture was left stirring for 10 minutes before $NaBH_4$ (0.222 g, 2 equiv.) was added in one portion. The reaction mixture was monitored by TLC and was completed within 1.5 hours. The reaction mixture was diluted with $CH_2Cl_2$, filtered over a plug of Celite®, and washed with $CH_2Cl_2$. The resulting filtrate was washed with brine (3 x 20 mL), dried over $MgSO_4$, filtered and concentrated *in vacuo* yielding the desired Compound **4** as a white solid (1.136 g, 80 %). **$^1$H-NMR** (400 MHz, Chloroform-*d*) δ (ppm) 7.81 (d, *J* = 2.1 Hz, 1H), 7.54 (dd, *J* = 8.6, 2.2 Hz, 1H), 7.36 (d, *J* = 8.6 Hz, 1H), 5.38 – 5.27 (m, 3H), 5.19 (d, *J* = 6.9 Hz, 1H), 4.73 (s, 2H), 4.20 (d, *J* = 8.8 Hz, 1H), 3.75 (s, 3H), 2.13 (s, 3H), 2.06 (s, 3H), 2.05 (s, 3H). **$^{13}$C-NMR** (101 MHz, Chloroform-*d*) δ (ppm) 170.2, 169.5, 169.5, 166.9, 148.3, 141.4, 137.4, 132.1, 123.4, 120.4, 100.0, 72.7, 71.2, 70.3, 68.9, 63.6, 53.2, 20.8, 20.7, 20.7. **HR-MS** (ESI): calcd. for $[C_{20}H_{23}NO_{13} + Na]^+$ 508.1061, found 508.1086. calcd. for $[C_{20}H_{23}NO_{13} + K]^+$ 524.0801, found 524.0810.

### 2.2.4 Synthesis of Compound 5

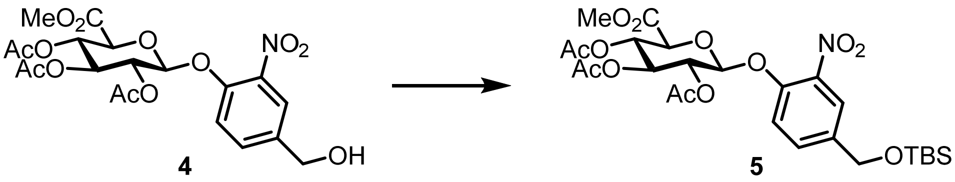


Compound **5** was synthesized similarly to the literature protocol[3]. Compound **4** (1.14 g, 1 equiv.) was added to a flame-dried flask and dissolved in *N,N*-dimethylformamide (DMF) (11.3 mL) under an atmosphere of argon. To that solution, imidazole (0.956 g, 6 equiv.) and 4-dimethylaminopyridine (DMAP) (0.0715 g, 0.25 equiv.) were added and stirred for 5 minutes. Thereafter, a solution of TBSCl (2.12 g, 6 equiv.) in DMF (8.0 mL) was added dropwise to the mixture. The reaction was left stirring at room temperature for 42 hours before full conversion was observed on TLC. The crude was diluted with $CH_2Cl_2$ and washed with $NH_4Cl$ (3x50 mL) and brine (2x50 mL). The organic layers were dried over $Na_2SO_4$, filtered, and the volume was reduced *in vacuo*. The crude compound was purified by silica column chromatography eluting with diethyl ether:pentane 40:60 to 60:40 affording Compound **5** (1.22 g, 86%). **$^1$H-NMR** (400 MHz, Chloroform-*d*) δ (ppm) 7.75 (d, *J* = 1.7 Hz, 1H), 7.49 (dd, , *J* = 9.2, 1.7 Hz 1H), 7.34 (d, *J* = 8.6 Hz, 1H), 5.38 – 5.28 (m, 3H), 5.17 (d, *J* = 6.7 Hz, 1H), 4.72 (s,

2H), 4.19 (d, $J$ = 8.7 Hz, 1H), 3.75 (s, 3H), 2.13 (s, 3H), 2.06 (s, 3H), 2.05 (s, 3H), 0.94 (s, 9H), 0.11 (s, 6H). **$^{13}$C-NMR** (101 MHz, Chloroform-*d*) δ (ppm) 170.20, 169.48, 169.46, 166.88, 147.93, 141.42, 138.24, 131.21, 122.57, 120.34, 100.22, 72.73, 71.35, 70.36, 68.96, 63.52, 53.22, 26.02, 20.77, 20.73, 20.68, 18.50, -5.17. **HR-MS** (ESI): calcd. for $[C_{26}H_{37}NO_{13}Si + Na]^+$ 622.1926, found 622.1941. calcd. for $[C_{26}H_{37}NO_{13}Si + K]^+$ 638.1666, found 638.1672.

**2.2.5 Synthesis of Compound 6**

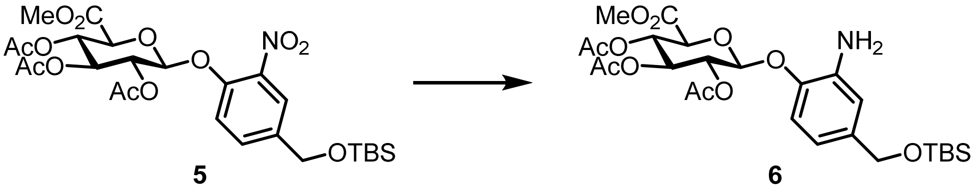


**5** **6**

Compound **6** was synthesized through a similar protocol as described in literature[3]. A suspension of Compound **5** (1.20 g, 1 equiv.) in abs. ethanol (75 mL) was prepared followed by purging with argon for 10 minutes. Pd/C (0.166 g, 0.7 equiv.) and ammonium formate (0.504 g, 4 equiv.) were added to the reaction mixture and the suspension was stirred for 3 hours until full conversion was observed on TLC. Upon completion, the mixture was filtered over a plug of Celite® followed by concentrating the filtrate under reduced pressure. The crude mixture was dissolved in ethyl acetate and washed with brine (3 x 20 mL), the organic phase was dried over $Na_2SO_4$, and filtered. The solvent was removed *in vacuo* to yield the pure Compound **6** (1.08 g, 95%). **$^1$H-NMR** (400 MHz, Chloroform-*d*) δ (ppm) 6.87 (d, $J$ = 8.2 Hz, 1H), 6.68 (d, $J$ = 1.6 Hz, 1H), 6.60 (dd, $J$ = 8.2, 1.7 Hz, 1H), 5.40 – 5.26 (m, 3H), 5.00 (d, $J$ = 7.3 Hz, 1H), 4.59 (s, 2H), 4.11 (d, $J$ = 7.2 Hz, 1H), 3.75 (s, 3H), 2.08 (s, 3H), 2.05 (s, 3H), 2.04 (s, 3H), 0.92 (s, 9H), 0.08 (s, 6H), residual EtOAc (4.12, 2.05, 1.26). **$^{13}$C-NMR** (101 MHz, Chloroform-*d*) δ (ppm) 170.2, 170.0, 169.6, 167.0, 143.5, 138.0, 137.7, 116.6, 116.0, 113.8, 101.0, 72.7, 71.8, 71.1, 69.4, 64.7, 53.2, 26.1, 20.9, 20.8, 20.7, 18.6, -5.1. **HR-MS** (ESI): $[C_{26}H_{39}NO_{11}Si+H]^+$ calcd. 570.2365, found 570.2381. $[C_{26}H_{39}NO_{11}Si+Na]^+$ calcd. 592.2184, found 592.2209.

**2.2.6 Synthesis of Compound 7**

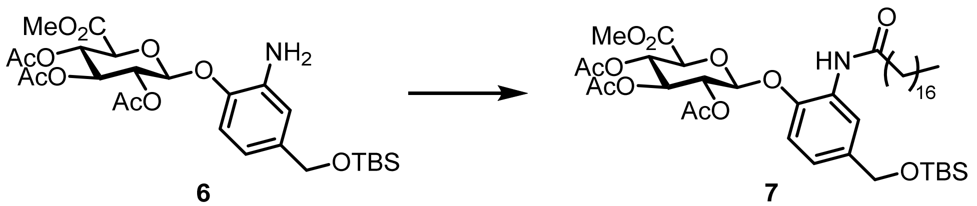


**6** **7**

Compound **6** (0.250 g, 1 equiv.) and triethyl amine (TEA) (0.122 mL, 2 equiv.) were added to a flame-dried flask and dissolved in dry $CH_2Cl_2$ (8 mL) under argon atmosphere. The mixture was cooled to 0°C and a solution of stearoyl chloride (0.146 g, 1.1 equiv.) in dry $CH_2Cl_2$ (4 mL) was

slowly added. The reaction was stirred for four hours at room temperature. Upon completion, the reaction was quenched with water (10 mL) and the compound was extracted using $CH_2Cl_2$ (3 x15 mL). The organic phases were dried over MgSO4, filtered and the solvent was removed under reduced pressure. The crude was purified by silica column chromatography eluting with 1:9 to 2:8 EtOAc:pen affording the desired Compound **7** (0.320 g, 87%). **$^1$H-NMR** (400 MHz, Chloroform-*d*) δ (ppm) 8.33 (d, *J* = 2.0 Hz, 1H), 7.86 (s, 1H), 7.05 (dd, *J* = 8.3, 2.0 Hz, 1H), 6.90 (d, *J* = 8.4 Hz, 1H), 5.42 – 5.27 (m, 3H), 5.04 (d, *J* = 7.5 Hz, 1H), 4.67 (s, 2H), 4.17 (d, *J* = 9.6 Hz, 1H), 3.75 (s, 3H), 2.40 (td, *J* = 7.4, 3.2 Hz, 2H), 2.08 (s, 3H), 2.06 (s, 3H), 2.05 (s, 3H), 1.74 – 1.67 (m, 2H), 1.39 – 1.24 (m, 28H), 0.92 (s, 9H), 0.87 (t, *J* = 6.8 Hz, 3H), 0.08 (s, 6H), residual EtOAc (4.12, 2.05, 1.26). **$^{13}$C-NMR** (101 MHz, $CDCl_3$) δ (ppm) 172.1, 170.2, 170.0, 169.5, 166.7, 144.1, 137.8, 129.2, 121.2, 118.4, 114.7, 100.5, 72.7, 71.3, 71.2, 69.4, 64.8, 60.5, 53.3, 37.9, 32.1, 29.9, 29.8, 29.7, 29.6, 29.5, 26.1, 25.8, 22.8, 21.2, 21.0, 20.7, 20.6, 18.6, 14.3, 14.3, -5.10. **HR-MS** (ESI): $[C_{44}H_{73}NO_{12}Si+Na]^+$ calcd. 858.4794, found 858.4811.

### 2.2.7 Synthesis of Compound 8

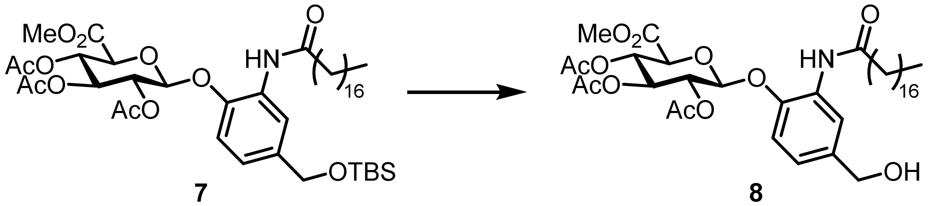


A solution was prepared of Compound **7** (0.320 g, 1 equiv.) in dry tetrahydrofurane (THF) (3.8 mL) in a flame-dried flask under and atmosphere of argon. The mixture was cooled to 0ºC and TEA·3HF (0.081 mL, 1.3 equiv.) in THF (1.25 mL) was slowly added. The resulting mixture was stirred at room temperature for 40.5 hours until completion. Then, the reaction mixture was diluted with $CH_2Cl_2$ (20 mL) and washed with sat. $NH_4Cl$ (2x10 mL) and brine (2x10 mL). The organic phase was dried over $MgSO_4$, filtered, and reduced *in vacuo*. The resulting crude was purified by silica column chromatography eluting with 4:6 to 5:5 EtOAc:pen to yield the pure Compound **8** (0.204 g, 74%). **$^1$H-NMR** (400 MHz, Chloroform-*d*) δ (ppm) 8.42 (d, *J* = 2.1 Hz, 1H), 7.89 (s, 1H), 7.06 (dd, *J* = 8.3, 2.1 Hz, 1H), 6.92 (d, *J* = 8.3 Hz, 1H), 5.43 – 5.28 (m, 3H), 5.06 (d, *J* = 7.5, 1H), 4.63 (s, 2H), 4.18 (d, *J* = 9.6 Hz, 1H), 3.76 (s, 3H), 2.42 (td, *J* = 7.4, 3.2 Hz, 2H), 2.08 (s, 3H), 2.07 (s, 3H), 2.06 (s, 3H), 1.75-1.68 (m, 2H), 1.42-1.25 (m, 28H), 0.88 (t, *J* = 6.8 Hz, 3H). **$^{13}$C-NMR** (101 MHz, Chloroform-*d*) δ (ppm) 172.3, 170.2, 170.0, 169.5, 166.6, 144.6, 137.2, 129.5, 122.3, 119.5, 114.9, 100.3, 72.7, 71.3, 71.2, 69.4, 65.1, 53.3, 37.8, 32.1, 29.9, 29.8, 29.8, 29.7, 29.6, 29.5, 29.5, 25.8, 22.8, 21.0, 20.7, 20.6, 14.3. **HR-MS** (ESI): $[C_{38}H_{59}NO_{12}+H]^+$ calcd. 722.4110, found 722.4112. $[C_{38}H_{59}NO_{12}+Na]^+$ calcd. 744.3929, found 744.3938.

### 2.2.8 Synthesis of Compound 9

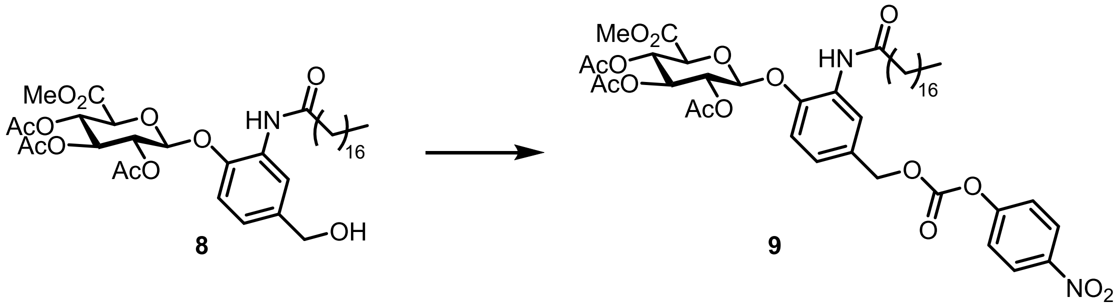


Compound **8** (0.197 g, 1 equiv.) and TEA (0.114 mL, 3 equiv.) were dissolved in dry $CH_2Cl_2$ (1.4 mL) in a flame-dried flask under an argon atmosphere. The solution was cooled to 0°C for 5 minutes followed by dropwise addition of a solution of 4-nitrophenyl chloroformate (0.077 g, 1.4 equiv.) in dry $CH_2Cl_2$ (2 mL). The resulting mixture was allowed to heat to room temperature and was left stirring for 21 hours before full conversion was observed by TLC. The crude mixture was directly absorbed onto Celite® and purified by silica column chromatography eluting with 2:8 to 3:7 EtOAc:pen affording the wanted Compound **9** (0.165 g, 68%). **$^1$H-NMR** (400 MHz, Chloroform-*d*) δ (ppm) 8.59 (d, *J* = 2.1 Hz, 1H), 8.27 (d, *J* = 9.1 Hz, 2H), 7.91 (s, 1H), 7.38 (d, *J* = 9.1 Hz, 2H), 7.09 (dd, *J* = 8.3, 1.9 Hz, 1H), 6.94 (d, *J* = 8.4 Hz, 1H), 5.46-5.27 (m, 3H), 5.22 (s, 2H), 5.09 (d, *J* = 7.5 Hz, 1H), 4.21 (d, *J* = 9.6 Hz, 1H), 3.76 (s, 3H), 2.44 (td, *J* = 7.5, 4.3 Hz, 2H), 2.09 (s, 3H), 2.08 (s, 3H), 2.06 (s, 3H), 1.76-1.68 (m, 2H), 1.41-1.19 (m, 28H) 0.88 (t, *J* = 6.9 Hz, 3H). **$^{13}$C-NMR** (101 MHz, Chloroform-*d*) δ (ppm) 172.4, 170.3, 169.9, 169.5, 166.6, 155.7, 152.5, 145.9, 145.3, 130.2, 129.7, 125.4, 123.9, 122.0, 121.0, 114.4, 99.9, 72.7, 71.2, 71.1, 70.8, 69.3, 53.4, 37.8, 32.1, 29.9, 29.8, 29.8, 29.7, 29.6, 29.5, 29.5, 25.8, 22.8, 21.0, 20.7, 20.6, 14.3. **HR-MS** (ESI): $[C_{45}H_{62}N_2O_{16}+Na]^+$ calcd. 909.3991, found 909.3999.

### 2.2.9 Synthesis of Compound 10

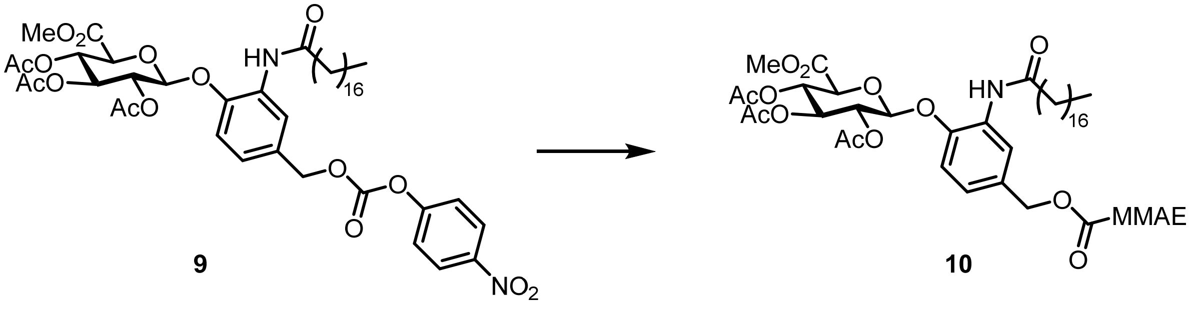


A solution of MMAE (0.095 g, 1.15 equiv.), HOBt (0.019 g, 1.1 equiv.) and TEA (0.024 mL, 1.5 equiv.) in dry DMF (1.0 mL) was prepared in a flame-dried flask under argon atmosphere. The solution was cooled to at 0ºC followed by dropwise addition of Compound **9** in DMF (1 mL). The

reaction mixture was left stirring at room temperature for 46 hours. Upon completion, the mixture was concentrated under reduced pressure and absorbed onto Celite®. The compound was purified by silica column chromatography eluting with 2% to 4% methanol in $CH_2Cl_2$ to yield the desired Compound **10** (0.145 g, 88%). **HR-MS** (ESI): $[C_{78}H_{124}N_6O_{20}+H]^+$ calcd. 1465.8943, found 1465.8973. $[C_{78}H_{124}N_6O_{20}+Na]^+$ calcd. 1487.8762, found 1487.8782. $[C_{78}H_{124}N_6O_{20}+2Na]^{2+}$ calcd. 755.4327, found 755.4325.

### 2.2.10 Synthesis of Compound 11

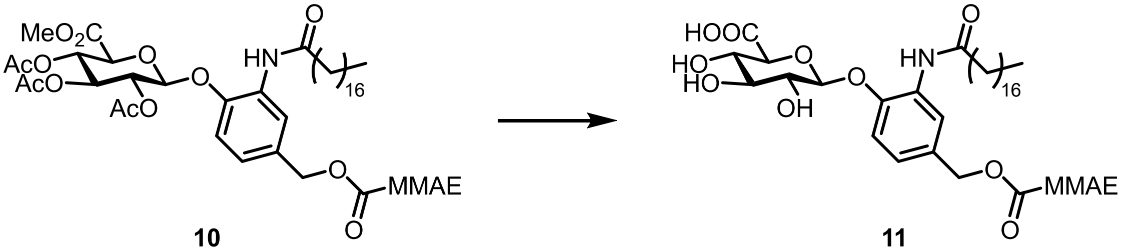


**10** **11**

Compound **10** (0.145 g, 1 equiv.) was mixed with dry methanol (6.0 mL) in a flame-dried flask under argon atmosphere. The suspension was cooled to 0ºC and NaOMe (0.3 equiv.) was slowly added. The reaction was allowed to heat to room temperature and followed by TLC. After 1 hour, full consumption of the starting material was still not achieved and additional NaOMe (0.3 equiv.) was added upon cooling. Addition of NaOMe (0.3 equiv.) was repeated three time in total before full conversion was observed on TLC. After completion, the reaction mixture was cooled to 0ºC and $H_2O$ (4.0 mL) and 2 M NaOH (60 uL) were slowly added. After 10 minutes, the mixture was neutralized with amberlite $120H^+$ ion exchange resin, filtered, and washed with $H_2O$ and methanol. The filtrate was lyophilized and the resulting crude was purified by silica column chromatography eluting with 2% to 10% methanol in $CH_2Cl_2$ followed by EtOAc:$CH_3CN$:MeOH:$H_2O$ 7:1:1:1 affording the pure, wanted Compound **11** (0.033 g, 25%). **HR-MS** (ESI): $[C_{71}H_{116}N_6O_{17}+H]^+$ calcd. 1325.8470, found 1325.8610. $[C_{71}H_{116}N_6O_{17}+Na]^+$ calcd. 1347.8289, found 1347.8446. $[C_{71}H_{116}N_6O_{17}+2Na]^{2+}$ calcd. 685.4091, found 685.3926. **HPLC**: $t_r$ (MMAE) = 11.294 min, $t_r$ (**11** without GUS) = 21.948 min, and $t_r$ (**11** with GUS) = 11.309 min.

### 2.2.11 Synthesis of FITC-cadaverine (Compound 12)

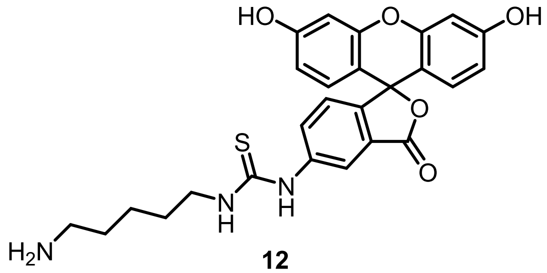


**12**

Fluorescein isothiocyanate (FITC) (0.020 g, 1 equiv.) was dissolved in a mixture of MeOH (0.5 mL) and DMF (0.3 mL) and added dropwise under cooling to a solution of cadaverine dihydrochloride (0.045 g, 5 equiv.) and TEA (0.052 g, 10 equiv.) in MeOH (0.3 mL). The reaction mixture was allowed to heat to room temperature and was left stirring for 5 hours. After a complete reaction, the solvent was evaporated under reduced pressure. The resulting crude was dissolved in DMSO and purified by preparative HPLC followed by lyophilization affording the desired Compound **12** (0.021 g, 81%). **$^1$H-NMR** (400 MHz, Methanol-d4) δ 8.23 – 8.13 (m, 1H), 7.74 (dd, *J* = 8.3, 2.1 Hz, 1H), 7.15 (d, *J* = 8.2 Hz, 1H), 6.85 (d, *J* = 8.8 Hz, 2H), 6.78 (d, *J* = 2.4 Hz, 2H), 6.64 (dd, *J* = 8.8, 2.4 Hz, 2H), 3.59 (t, J = 7.1 Hz, 2H), 2.90 (t, J = 7.7 Hz, 2H), 1.78 – 1.59 (m, 4H), 1.44 (q, J = 8.1 Hz, 2H). **HR-MS** (ESI): $[C_{26}H_{25}N_3O_5S+H]^+$ calcd. 492.1588, found 492.1595. $[C_{26}H_{25}N_3O_5S+Na]^+$ calcd. 514.1407, found 514.1402.

**2.2.12 Fluorescent labelling of Glu-C18-MMAE and Glu-MMAE (Compounds 13 and 14).**
The compounds Glu-MMAE and Glu-C18-MMAE were labeled using FITC-cadaverine (compound **12**). The reaction was performed as a coupling between the carboxylic acid of glucuronic acid and the amine of FITC-cadaverine. Glu-MMAE or Glu-C18-MMAE (1 mM), (1-[Bis(dimethylamino)methylene]-1H-1,2,3-triazolo[4,5-b]pyridinium 3-oxide hexafluorophosphate (HATU) (1.5 mM), and *N*,*N*-diisopropylethylamine (3 mM) were dissolved in DMF. After 10 minutes, compound **12** (1.2 mM) was added to the mixture and the reaction was left overnight in the dark. The crude mixture was analyzed with analytical HPLC (**Figure S2**) and HR-MS to determine the formation of the wanted products **13** and **14**. The crude mixture was directly used for visualization in GUVs and cell culture.

Compound **13**:

**HR-MS** (ESI): $[C_{79}H_{104}N_8O_{20}S+H]^+$ calcd. 1517.7161, found 1517.7209. $[C_{79}H_{104}N_8O_{20}S+Na]^+$ calcd. 1539.6980, found 1539.7025.

Compound **14**:

**HR-MS** (ESI): $[C_{97}H_{139}N_9O_{21}S+H+Na]^{2+}$ calcd. 910.9885, found 910.9818.

**HR-MS** (ESI): $[C_{97}H_{139}N9O21S-H]^-$ calcd. 1796.9733, found 1796.9571.

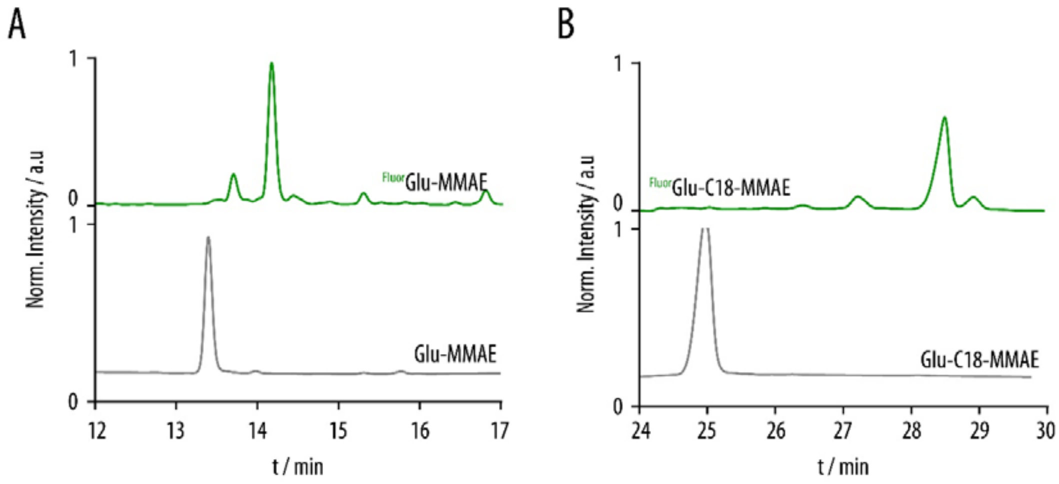


**Figure S2.** A) HPLC traces of the labelling reaction of Glu-MMAE with **12** (top) and control Glu-MMAE (bottom). B) HPLC traces of the labelling reaction of Glu-C18-MMAE with **12** (top) and control Glu-C18-MMAE (bottom).

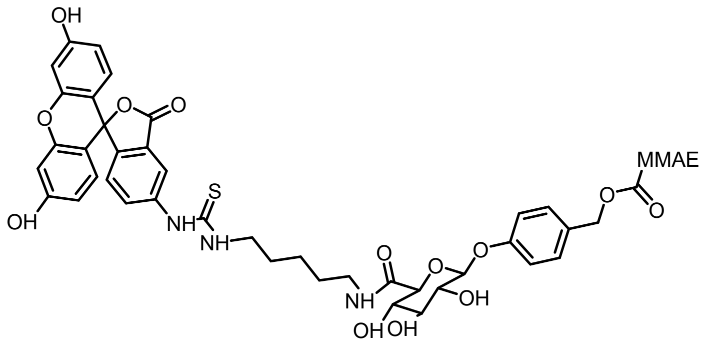
OH
O
O
O
OH
S
NH
NH
MMAE
O
O
O
NH
O
O
OH
OH
OH
O

**13**

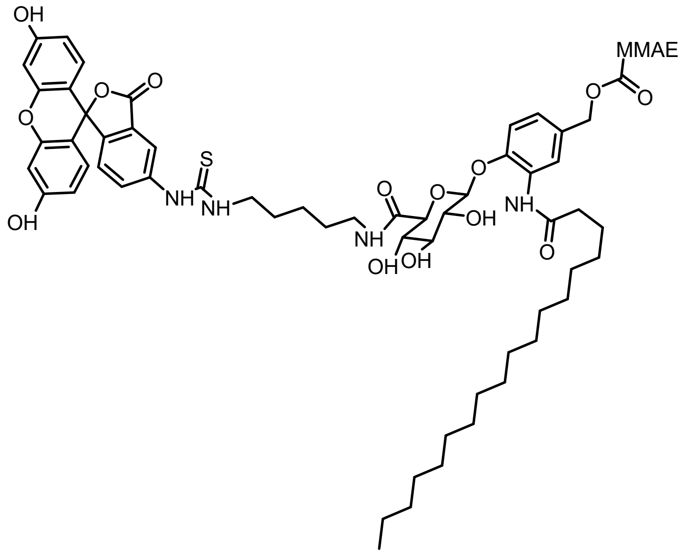
OH
O
O
O
OH
S
NH
NH
MMAE
O
O
O
NH
O
O
OH
OH
OH
NH
O

**14**

## 2.3 NMR spectra

Below is $^{1}$H-NMR and $^{13}$C-NMR for compound **2-9** and compound **12.**

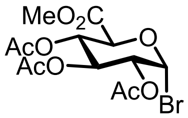


**2**

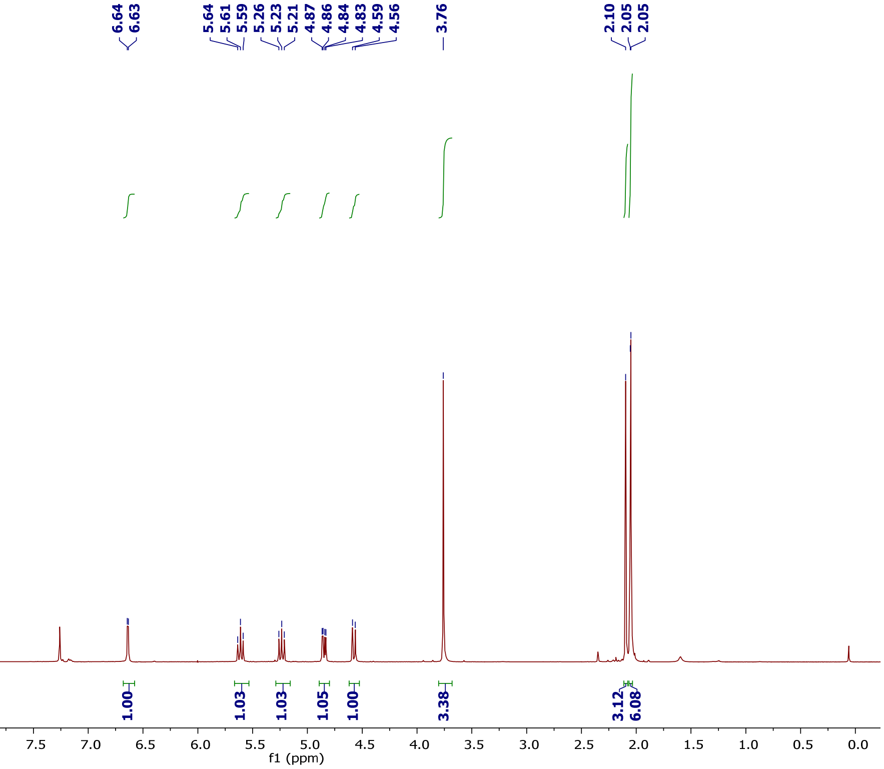


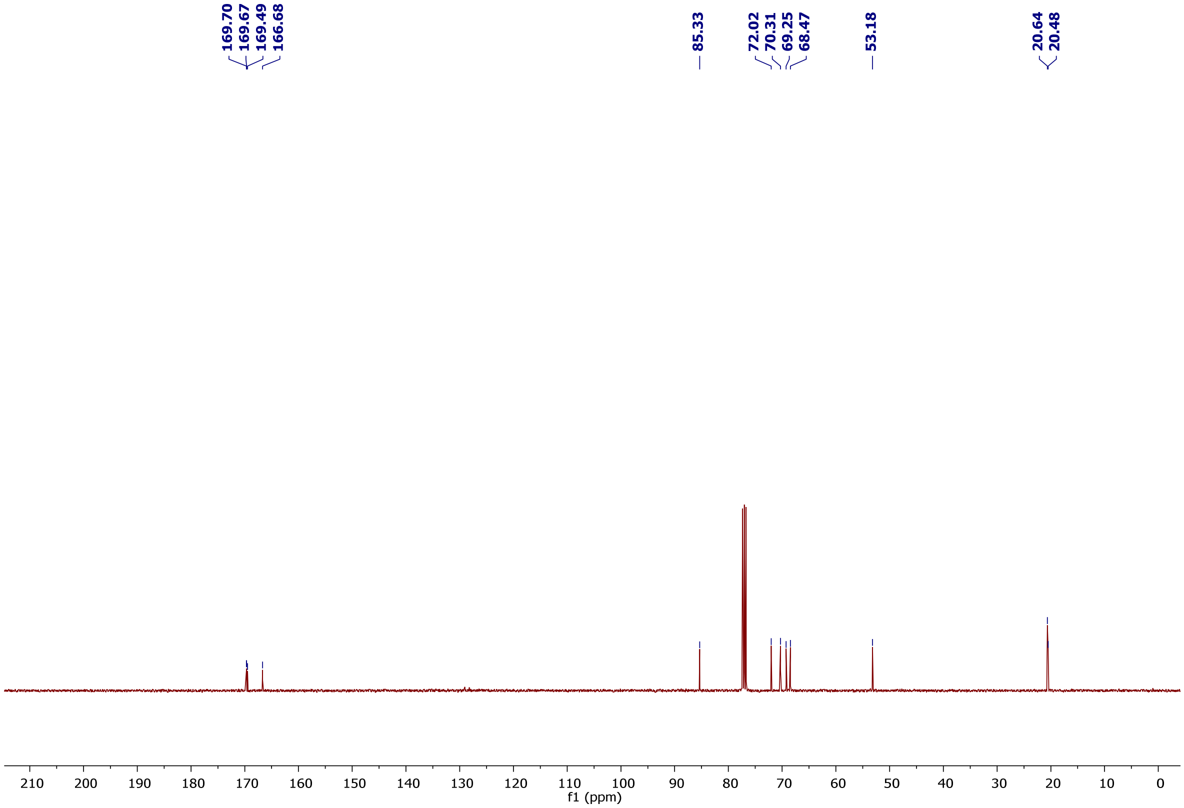

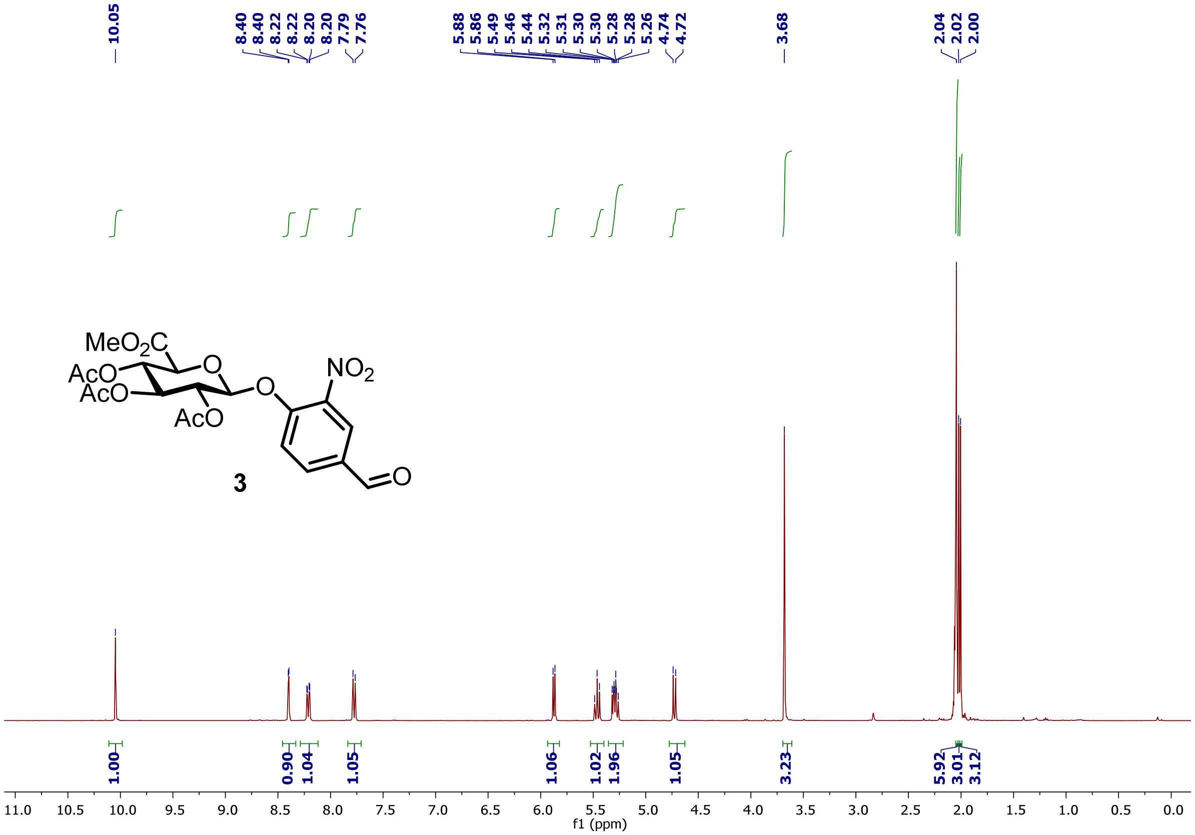
10.05
8.40
8.40
8.22
8.22
8.20
8.20
7.79
7.76
5.88
5.86
5.49
5.46
5.44
5.32
5.31
5.30
5.30
5.28
5.28
5.26
4.74
4.72
3.68
2.04
2.02
2.00
MeO2C
AcO
AcO
AcO
O
O
NO2
O
3
1.00
0.90
1.04
1.05
1.06
1.02
1.96
1.05
3.23
5.92
3.01
3.12
11.0 10.5 10.0 9.5 9.0 8.5 8.0 7.5 7.0 6.5 6.0 5.5 5.0 4.5 4.0 3.5 3.0 2.5 2.0 1.5 1.0 0.5 0.0
f1 (ppm)

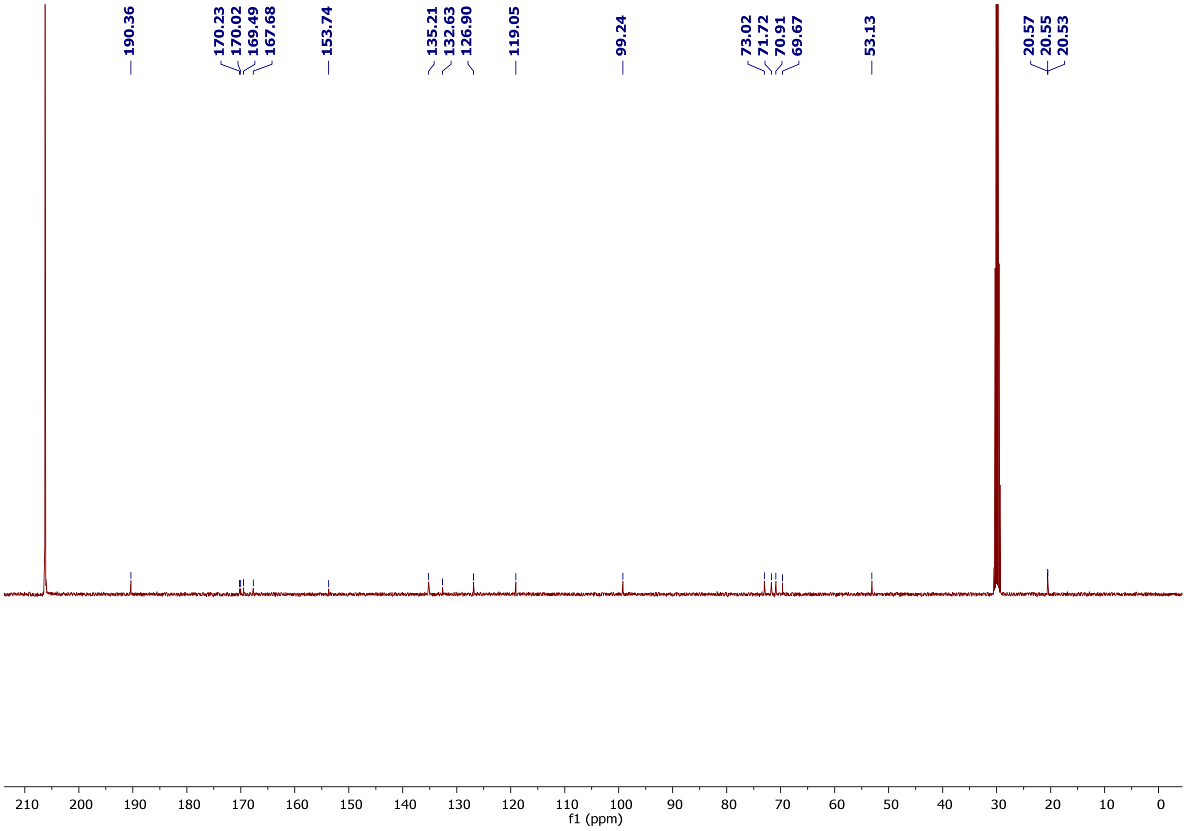
190.36
170.23
170.02
169.49
167.68
153.74
135.21
132.63
126.90
119.05
99.24
73.02
71.72
70.91
69.67
53.13
20.57
20.55
20.53
210 200 190 180 170 160 150 140 130 120 110 100 90 80 70 60 50 40 30 20 10 0
f1 (ppm)

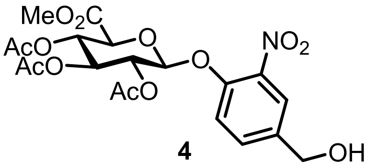
MeO2C
AcO
AcO
AcO
O
O
NO2
OH

**4**

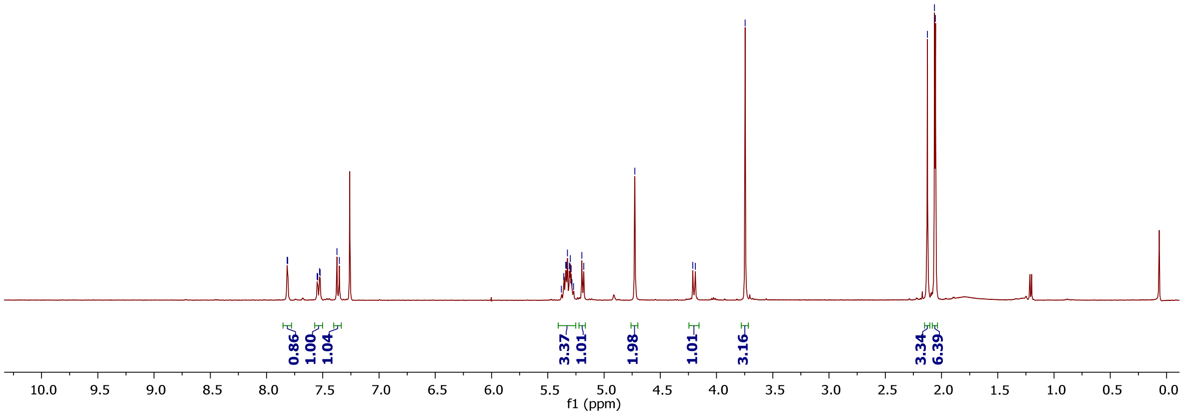
7.82
7.81
7.55
7.55
7.53
7.52
7.37
7.35
5.38
5.36
5.34
5.33
5.33
5.31
5.30
5.29
5.29
5.27
5.20
5.18
4.73
4.21
4.19
3.75
2.13
2.06
2.05
0.86
1.00
1.04
3.37
1.01
1.98
1.01
3.16
3.34
6.39
10.0 9.5 9.0 8.5 8.0 7.5 7.0 6.5 6.0 5.5 5.0 4.5 4.0 3.5 3.0 2.5 2.0 1.5 1.0 0.5 0.0
f1 (ppm)

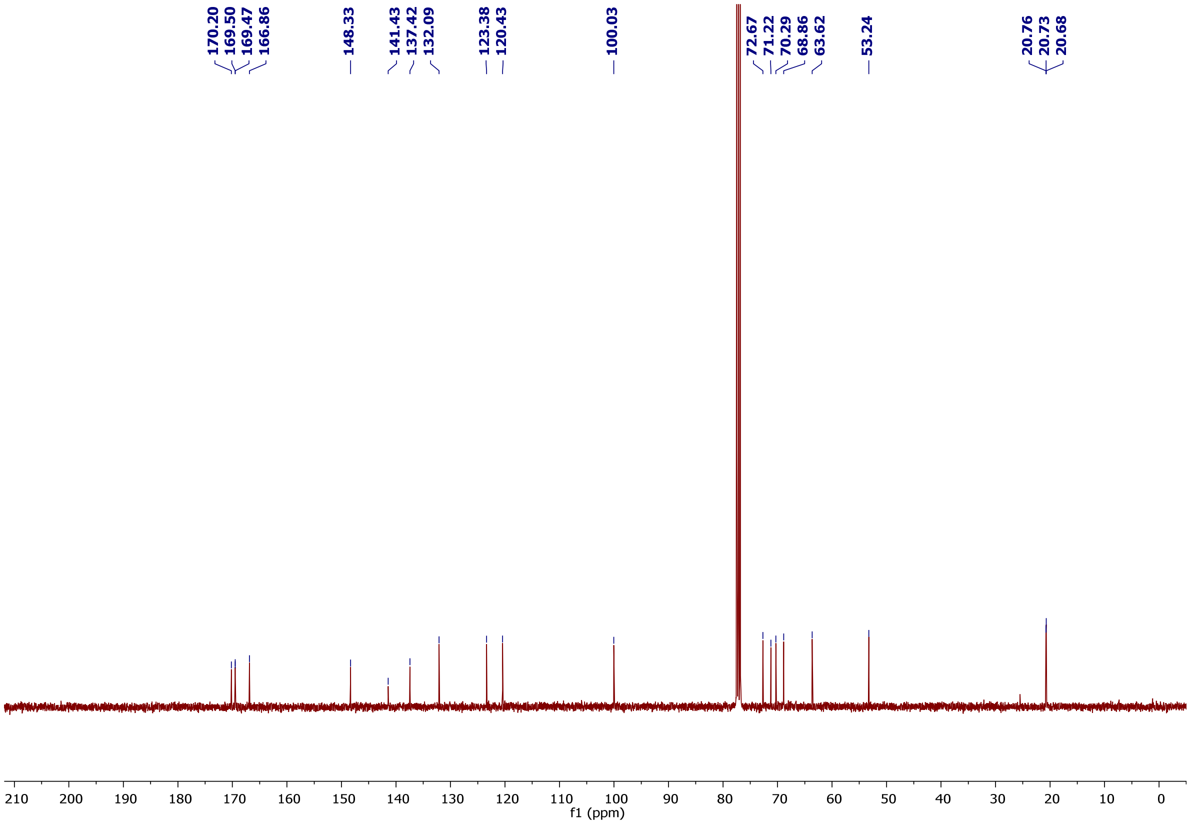
170.20
169.50
169.47
166.86
148.33
141.43
137.42
132.09
123.38
120.43
100.03
72.67
71.22
70.29
68.86
63.62
53.24
20.76
20.73
20.68
210 200 190 180 170 160 150 140 130 120 110 100 90 80 70 60 50 40 30 20 10 0
f1 (ppm)

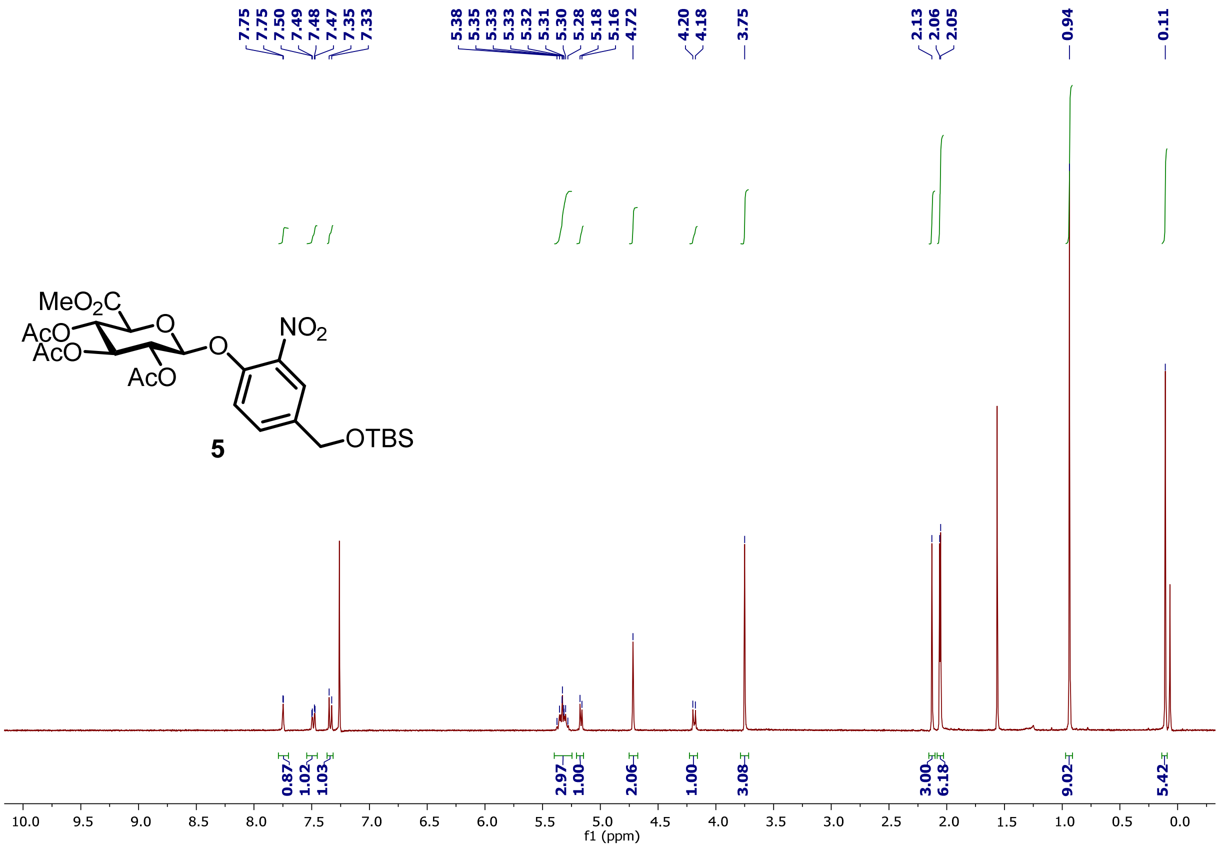
7.75 7.75 7.50 7.49 7.48 7.47 7.35 7.33
5.38 5.35 5.33 5.33 5.32 5.31 5.30 5.28 5.18 5.16 4.72
4.20 4.18
3.75
2.13 2.06 2.05
0.94
0.11
MeO2C
AcO
AcO
AcO
O
O
NO2
OTBS
5
0.87 1.02 1.03 2.97 1.00 2.06 1.00 3.08 3.00 6.18 9.02 5.42
10.0 9.5 9.0 8.5 8.0 7.5 7.0 6.5 6.0 5.5 5.0 4.5 4.0 3.5 3.0 2.5 2.0 1.5 1.0 0.5 0.0
f1 (ppm)

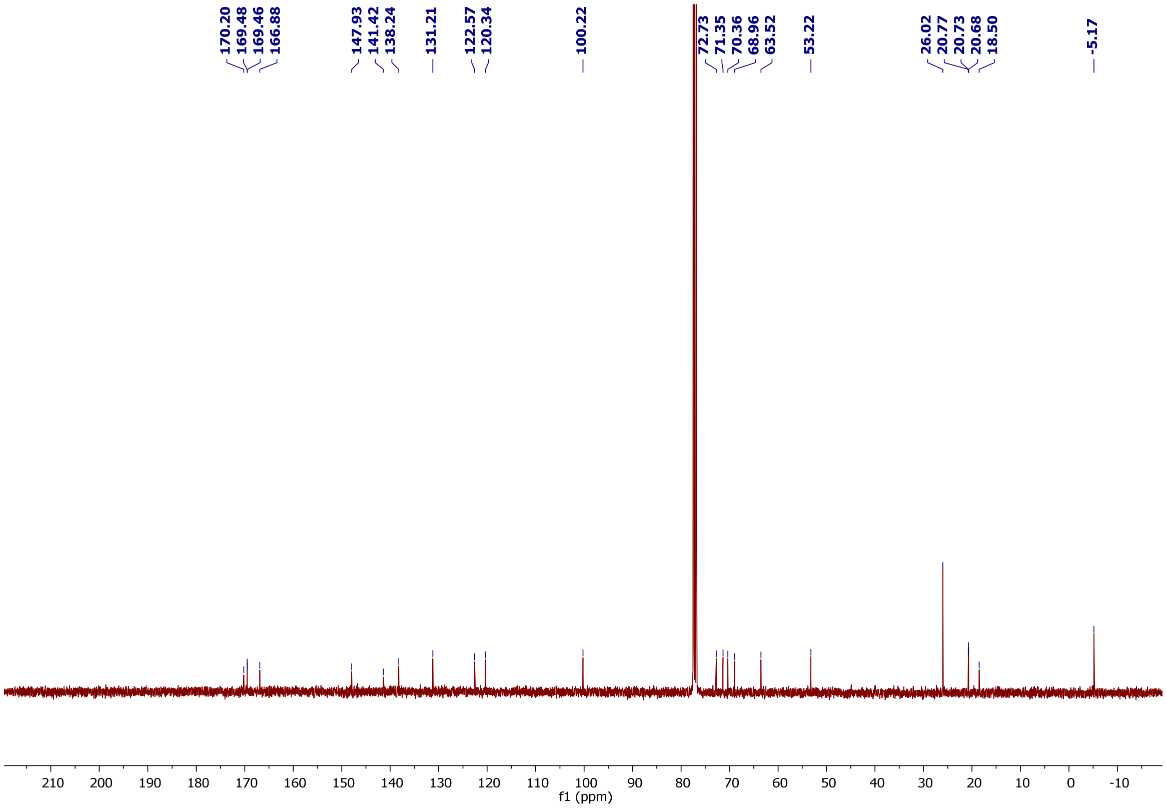
170.20 169.48 169.46 166.88
147.93 141.42 138.24
131.21
122.57 120.34
100.22
72.73 71.35 70.36 68.96 63.52
53.22
26.02 20.77 20.73 20.68 18.50
-5.17
210 200 190 180 170 160 150 140 130 120 110 100 90 80 70 60 50 40 30 20 10 0 -10
f1 (ppm)

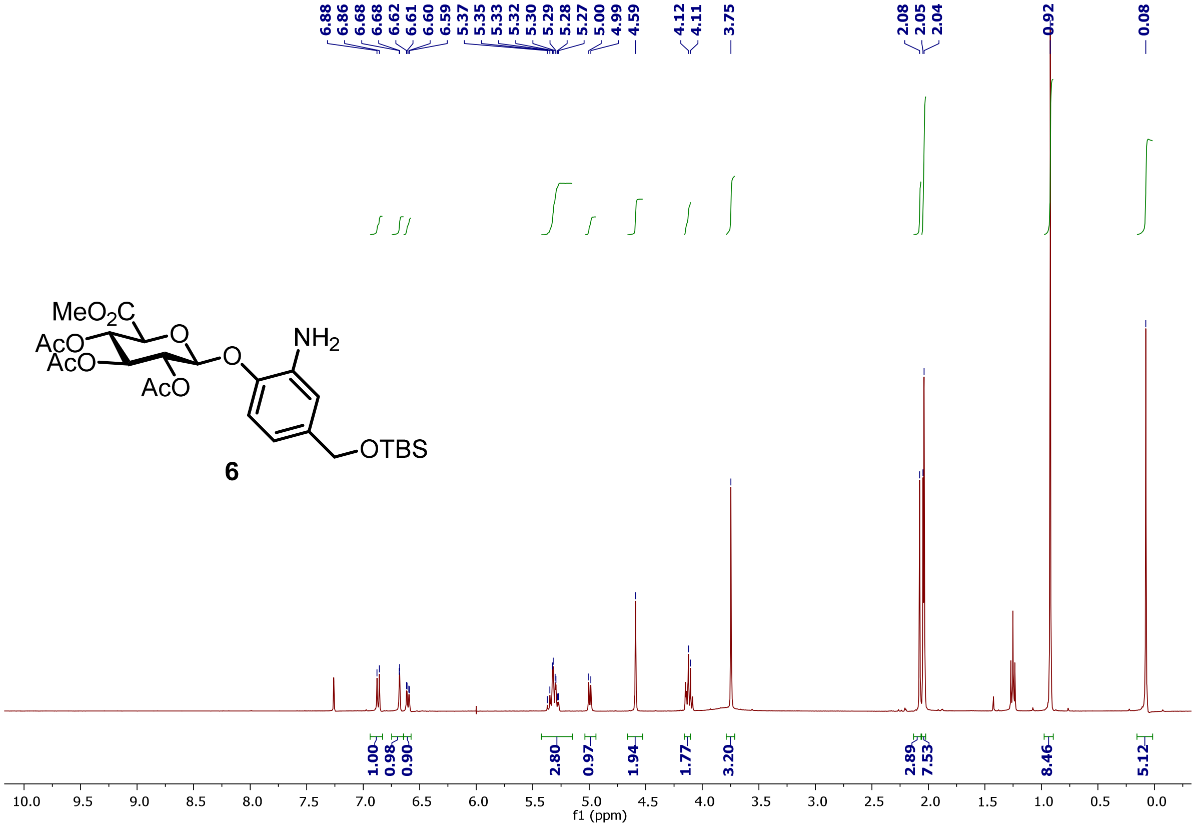
6.88
6.86
6.68
6.68
6.62
6.61
6.60
6.59
5.37
5.35
5.33
5.32
5.30
5.29
5.28
5.27
5.00
4.99
4.59
4.12
4.11
3.75
2.08
2.05
2.04
0.92
0.08
MeO2C
AcO
AcO
AcO
O
O
NH2
OTBS
6
1.00
0.98
0.90
2.80
0.97
1.94
1.77
3.20
2.89
7.53
8.46
5.12
f1 (ppm)

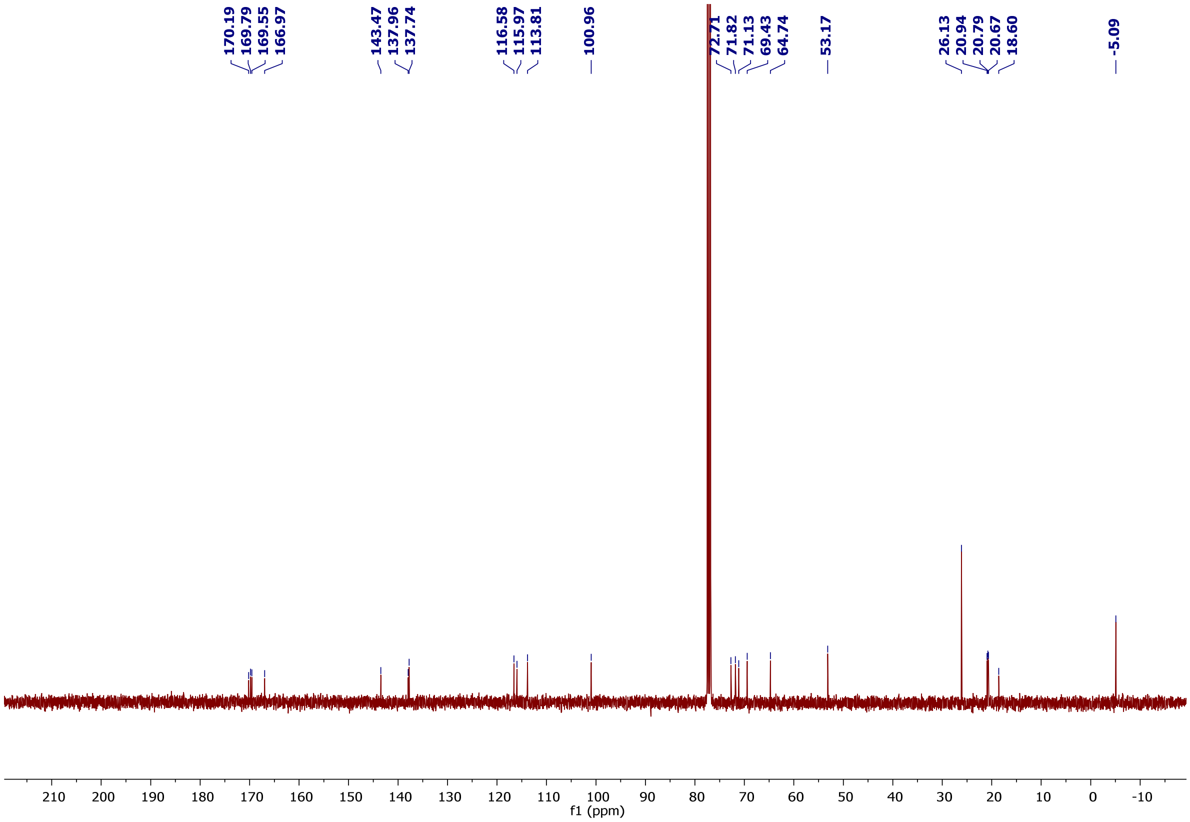
170.19
169.79
169.55
166.97
143.47
137.96
137.74
116.58
115.97
113.81
100.96
72.71
71.82
71.13
69.43
64.74
53.17
26.13
20.94
20.79
20.67
18.60
-5.09
f1 (ppm)

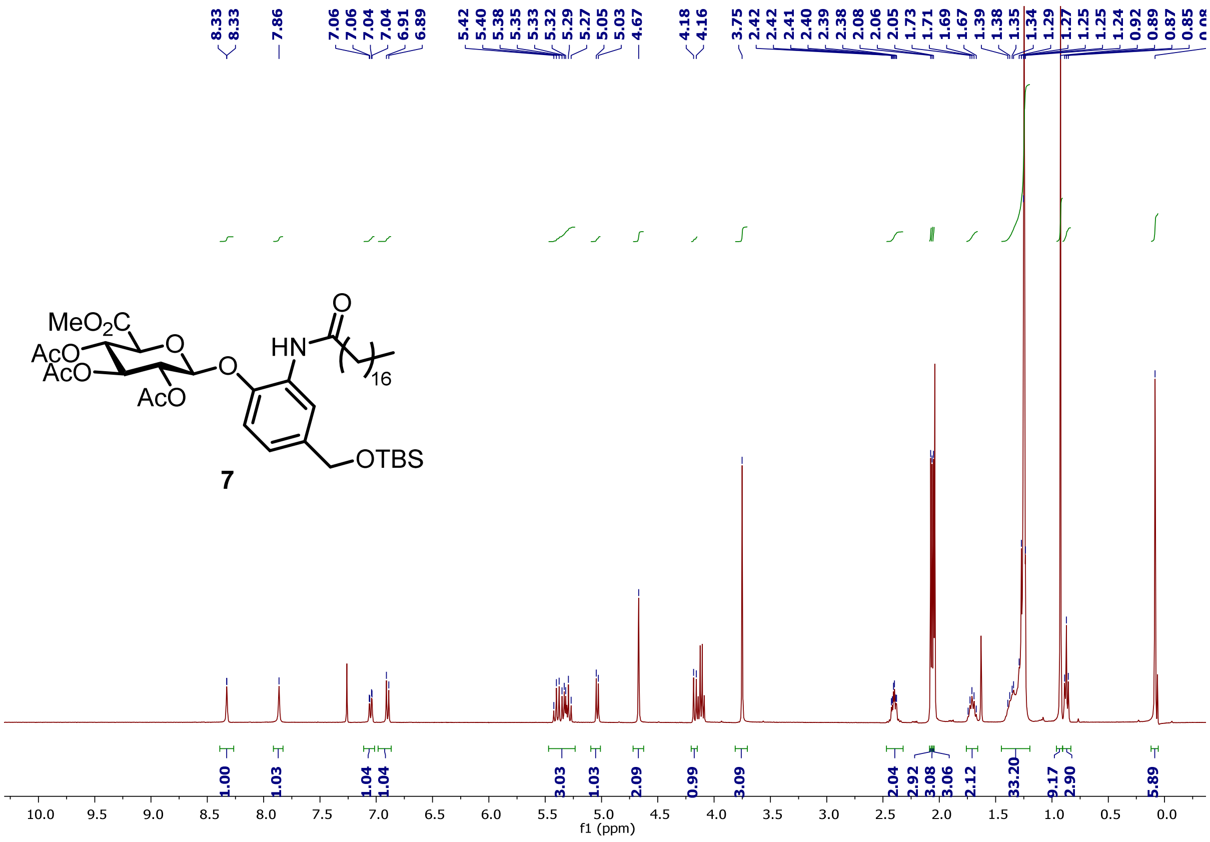
8.33 8.33 7.86 7.06 7.06 7.04 7.04 6.91 6.89 5.42 5.40 5.38 5.35 5.33 5.32 5.29 5.27 5.05 5.03 4.67 4.18 4.16 3.75 2.42 2.42 2.41 2.40 2.39 2.38 2.08 2.06 2.05 1.73 1.71 1.69 1.67 1.39 1.38 1.35 1.34 1.29 1.27 1.25 1.25 1.24 0.92 0.89 0.87 0.85
MeO2C
AcO
AcO
AcO
O
HN
16
OTBS
7
1.00 1.03 1.04 1.04 3.03 1.03 2.09 0.99 3.09 2.04 2.92 3.08 3.06 2.12 33.20 9.17 2.90 5.89
f1 (ppm)

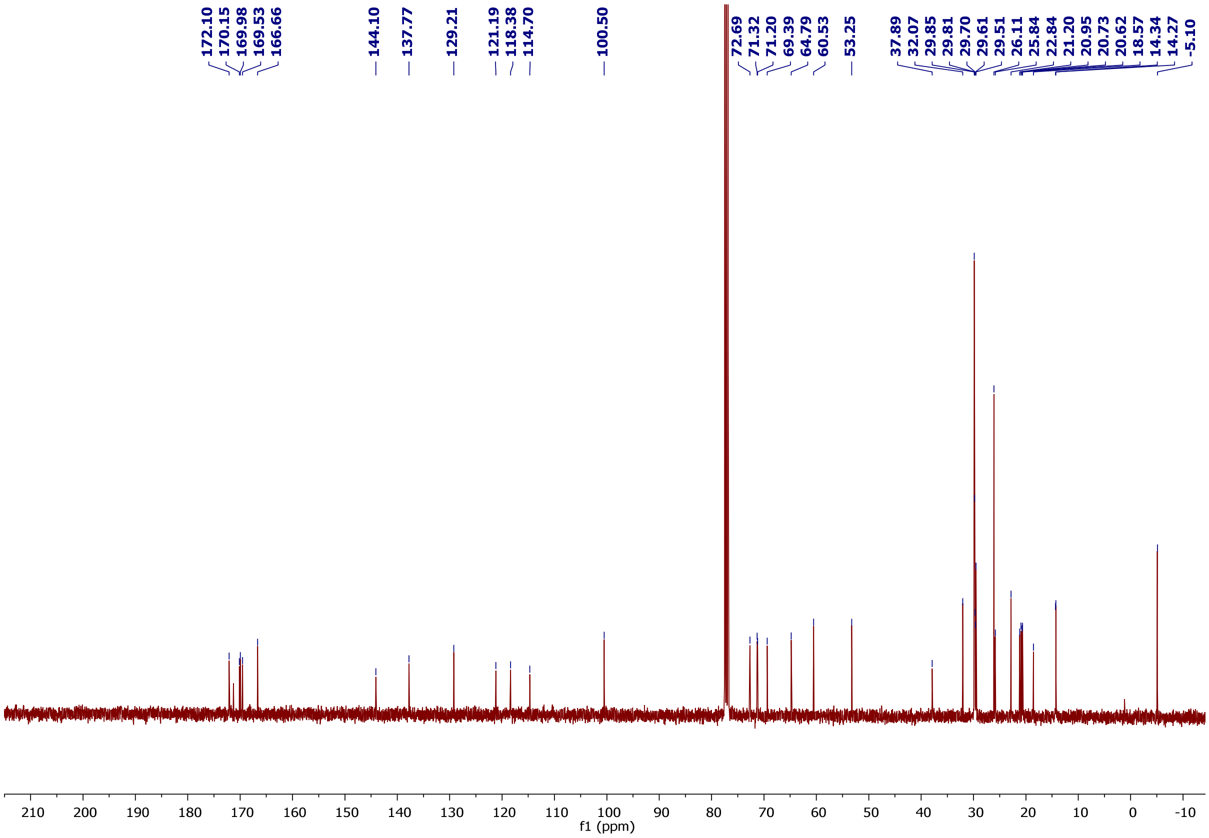
172.10 170.15 169.98 169.53 166.66 144.10 137.77 129.21 121.19 118.38 114.70 100.50 72.69 71.32 71.20 69.39 64.79 60.53 53.25 37.89 32.07 29.85 29.81 29.70 29.61 29.51 26.11 25.84 22.84 21.20 20.95 20.73 20.62 18.57 14.34 14.27 -5.10
f1 (ppm)

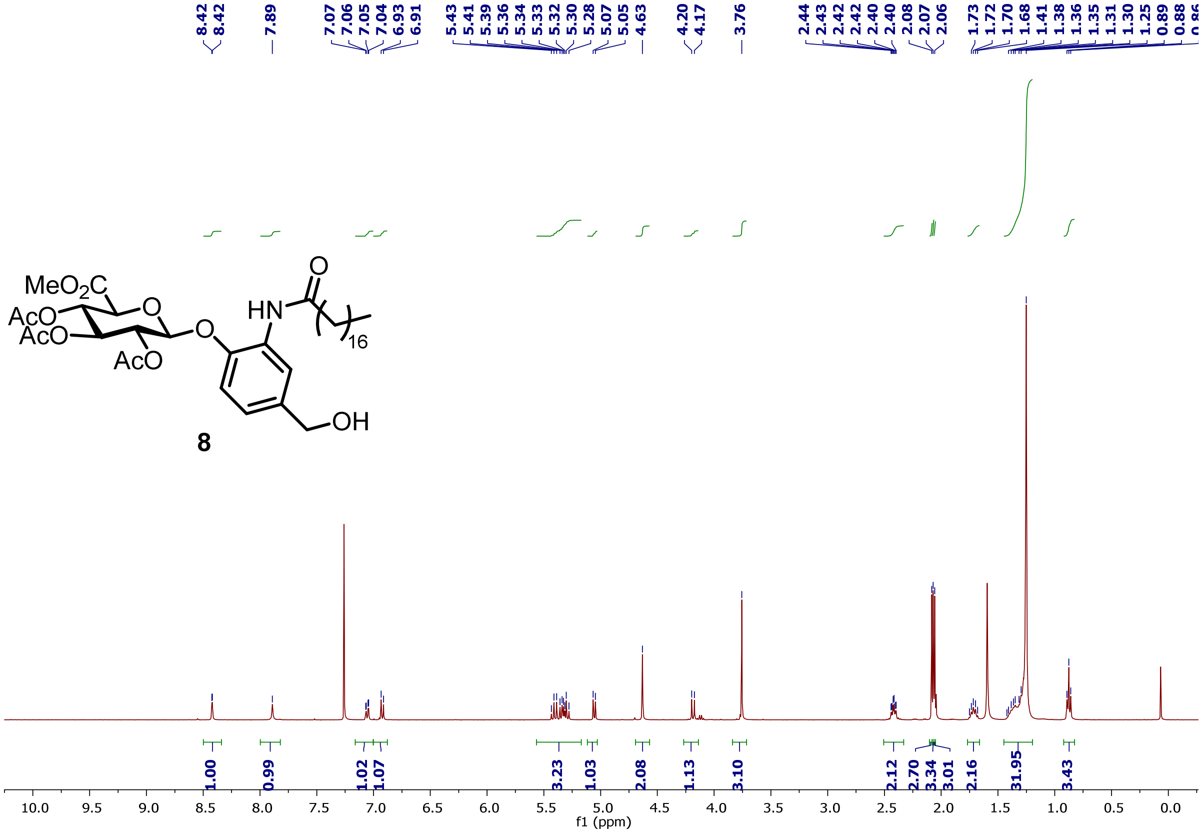
8.42 8.42 7.89 7.07 7.06 7.05 7.04 6.93 6.91 5.43 5.41 5.39 5.36 5.34 5.33 5.32 5.30 5.28 5.07 5.05 4.63 4.20 4.17 3.76 2.44 2.43 2.42 2.42 2.40 2.40 2.08 2.07 2.06 1.73 1.72 1.70 1.68 1.41 1.38 1.36 1.35 1.31 1.30 1.25 0.89 0.88
MeO2C
AcO
AcO
AcO
O
O
HN
O
16
OH
8
1.00 0.99 1.02 1.07 3.23 1.03 2.08 1.13 3.10 2.12 2.70 3.34 3.01 2.16 31.95 3.43
f1 (ppm)

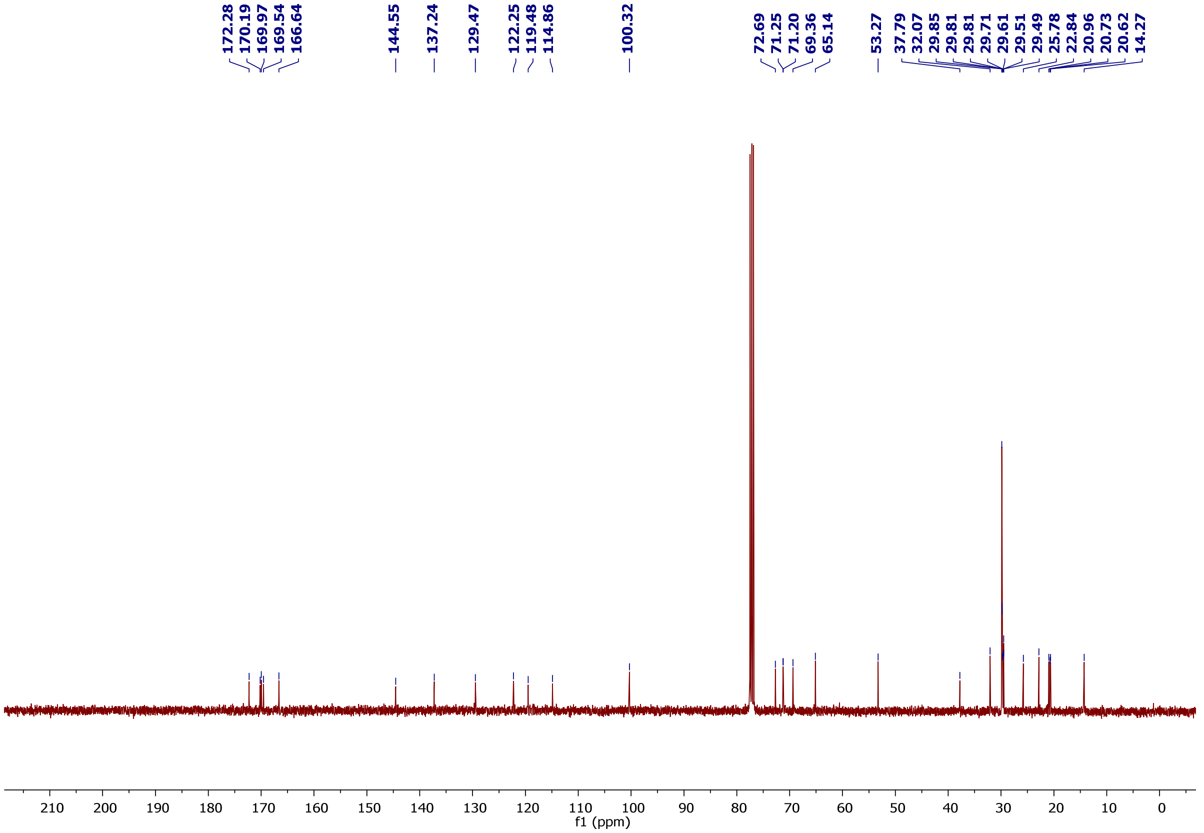
172.28 170.19 169.97 169.54 166.64 144.55 137.24 129.47 122.25 119.48 114.86 100.32 72.69 71.25 71.20 69.36 65.14 53.27 37.79 32.07 29.85 29.81 29.71 29.61 29.51 29.49 25.78 22.84 20.96 20.73 20.62 14.27
f1 (ppm)

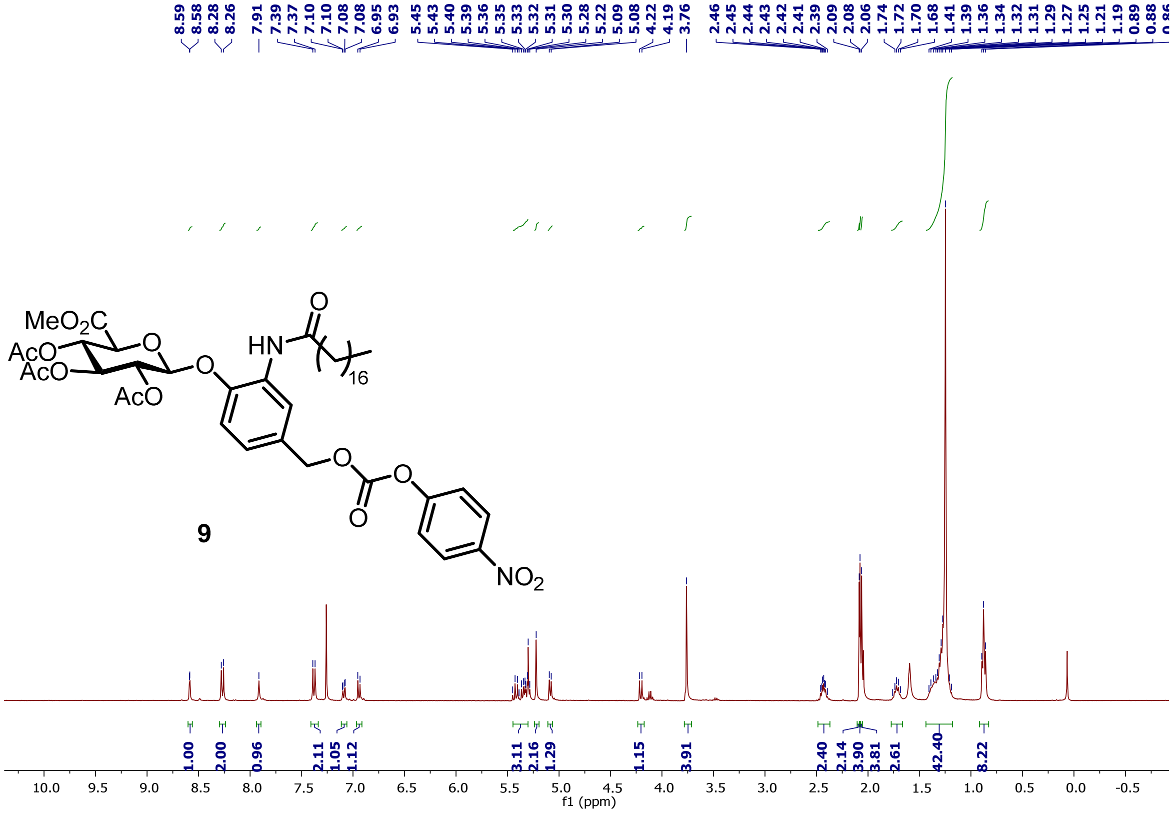

8.59 8.58 8.28 8.26 7.91 7.39 7.37 7.10 7.10 7.08 7.08 6.95 6.93 5.45 5.43 5.40 5.39 5.36 5.35 5.33 5.32 5.31 5.30 5.28 5.22 5.09 5.08 4.22 4.19 3.76 2.46 2.45 2.44 2.43 2.42 2.41 2.39 2.09 2.08 2.06 1.74 1.72 1.70 1.68 1.41 1.39 1.36 1.34 1.32 1.31 1.29 1.27 1.25 1.21 1.19 0.89 0.88
MeO2C
AcO
AcO
AcO
HN
16
NO2
9
1.00 2.00 0.96 2.11 1.05 1.12 3.11 2.16 1.29 1.15 3.91 2.40 2.14 3.90 3.81 2.61 42.40 8.22
10.0 9.5 9.0 8.5 8.0 7.5 7.0 6.5 6.0 5.5 5.0 4.5 4.0 3.5 3.0 2.5 2.0 1.5 1.0 0.5 0.0 -0.5
f1 (ppm)


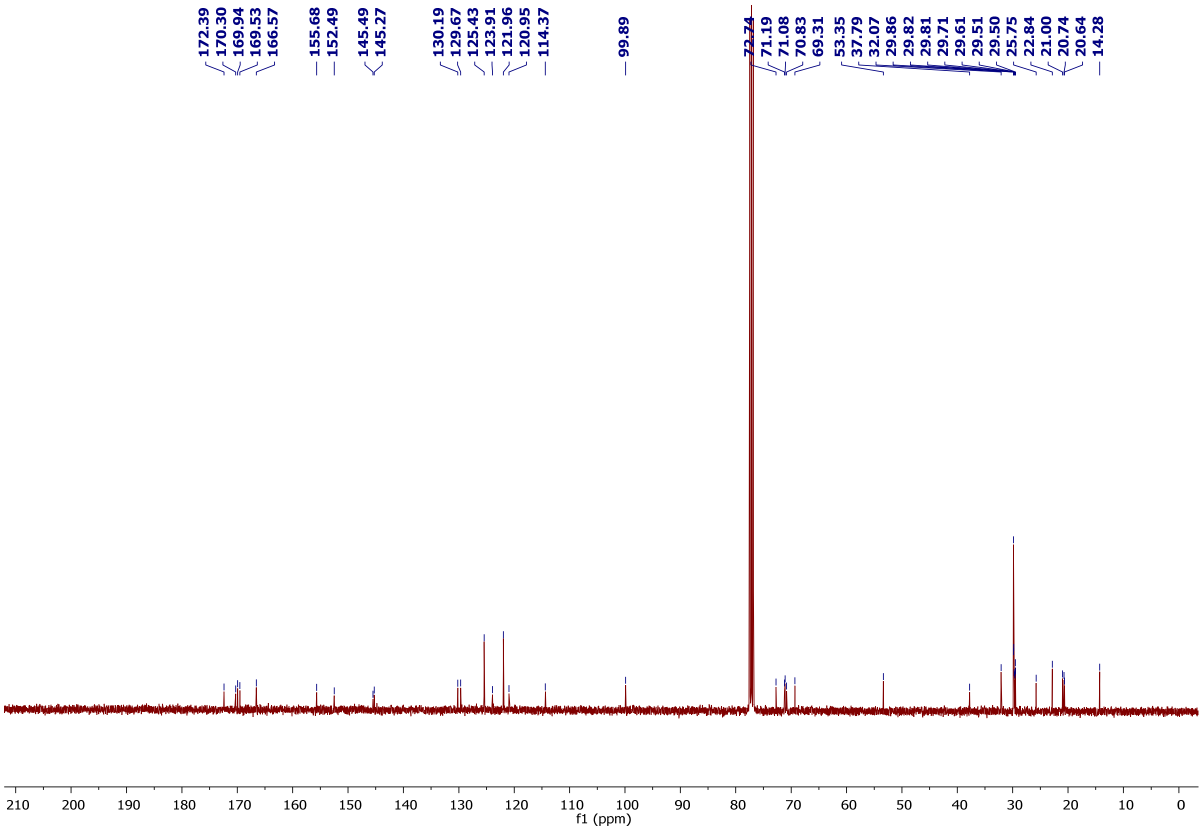

172.39 170.30 169.94 169.53 166.57 155.68 152.49 145.49 145.27 130.19 129.67 125.43 123.91 121.96 120.95 114.37 99.89 71.19 71.08 70.83 69.31 53.35 37.79 32.07 29.86 29.82 29.81 29.71 29.61 29.51 29.50 25.75 22.84 21.00 20.74 20.64 14.28
210 200 190 180 170 160 150 140 130 120 110 100 90 80 70 60 50 40 30 20 10 0
f1 (ppm)

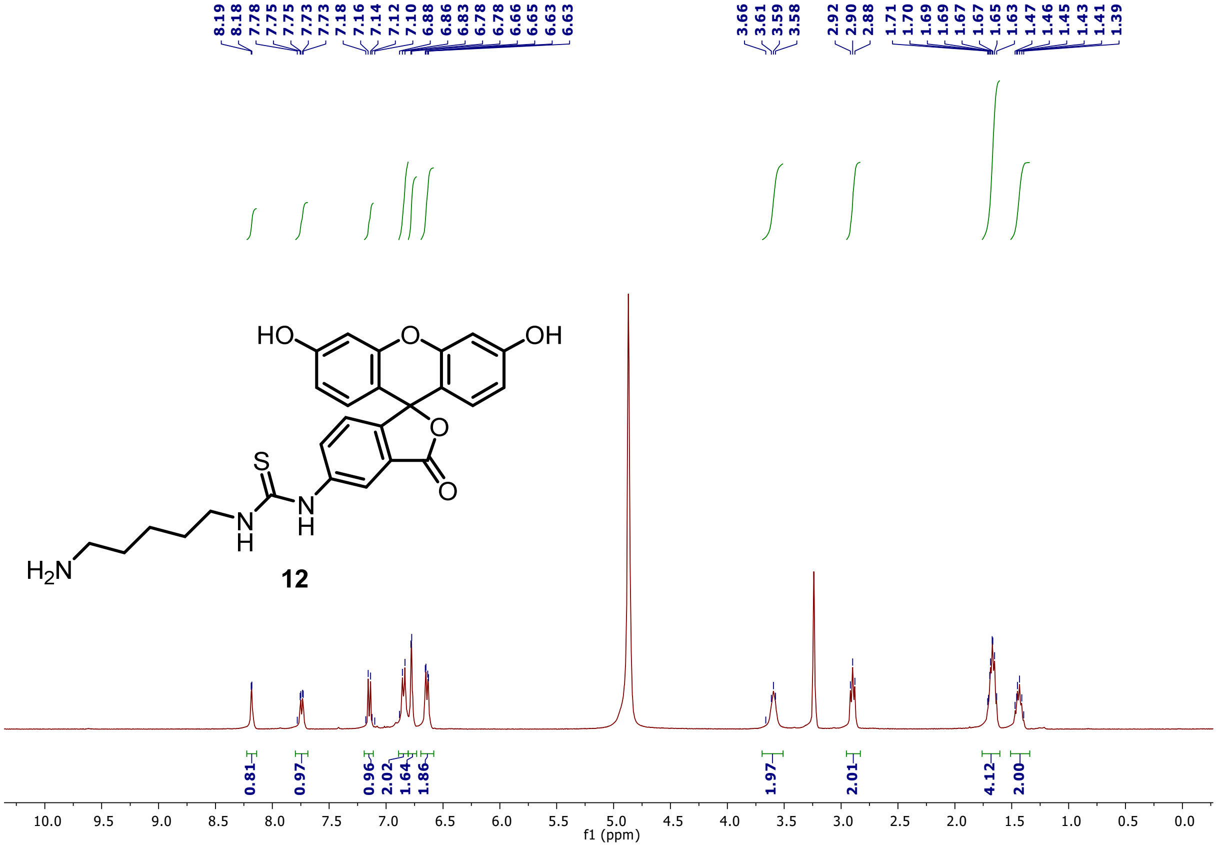

## 2.4 MS spectra

MS spectra for compound **10-11** and **13-14**.

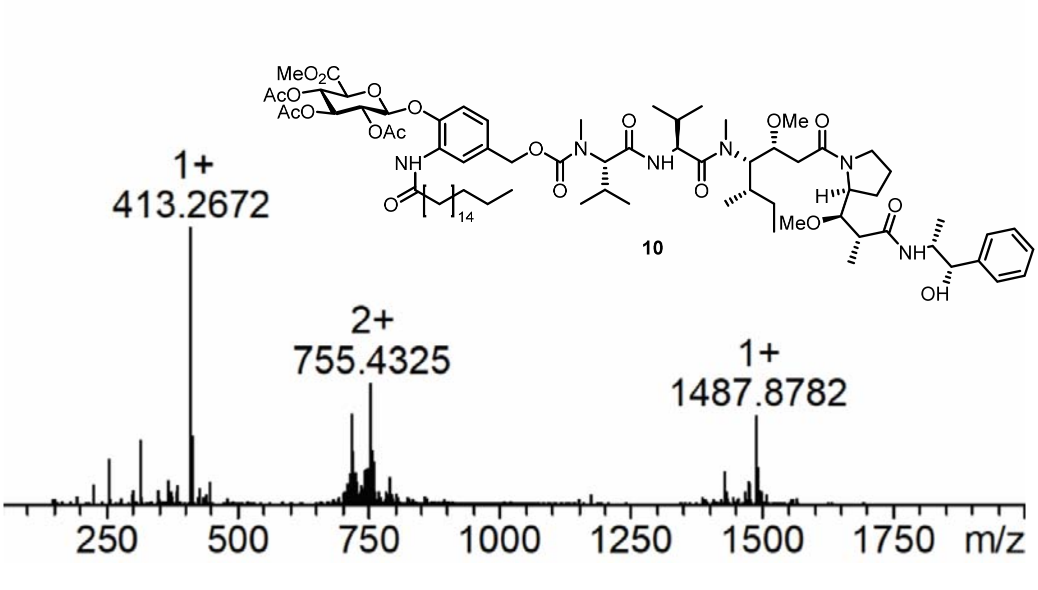

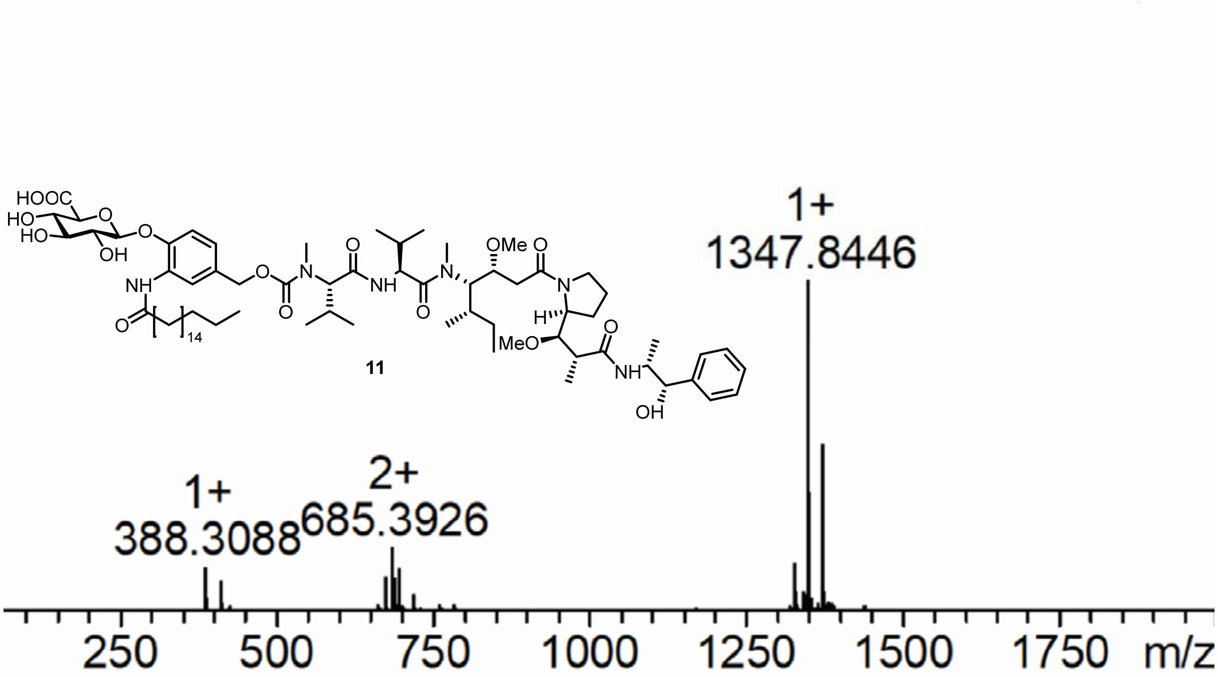
1+
1347.8446
1+
388.3088
2+
685.3926
11
250
500
750
1000
1250
1500
1750
m/z

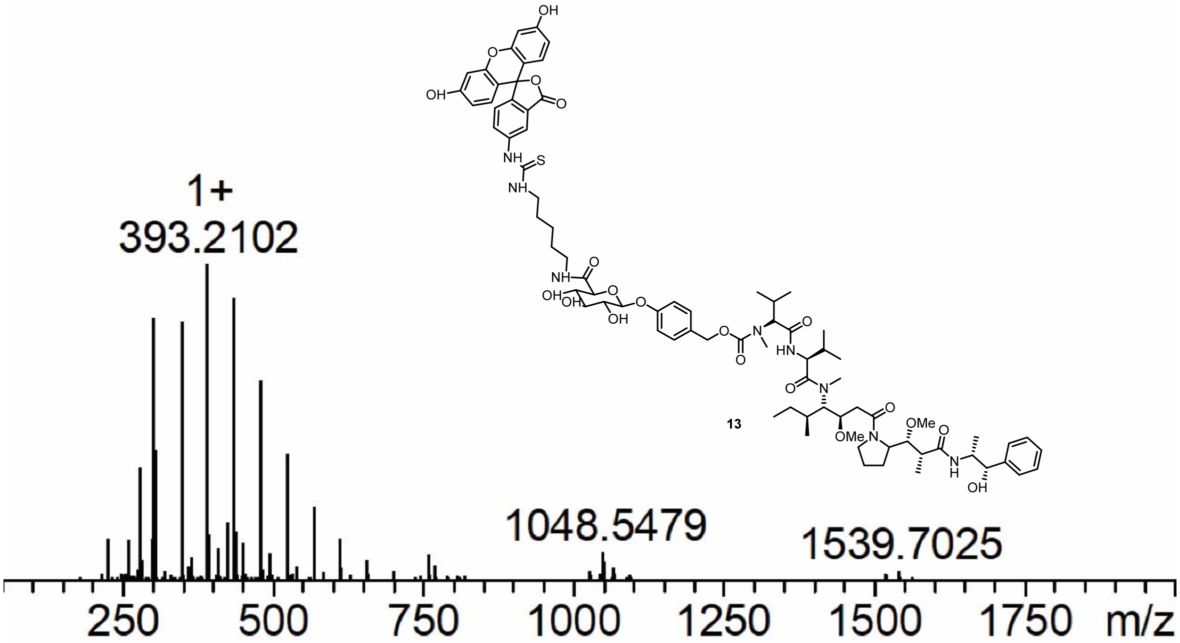
1+
393.2102
1048.5479
1539.7025
13
250
500
750
1000
1250
1500
1750
m/z

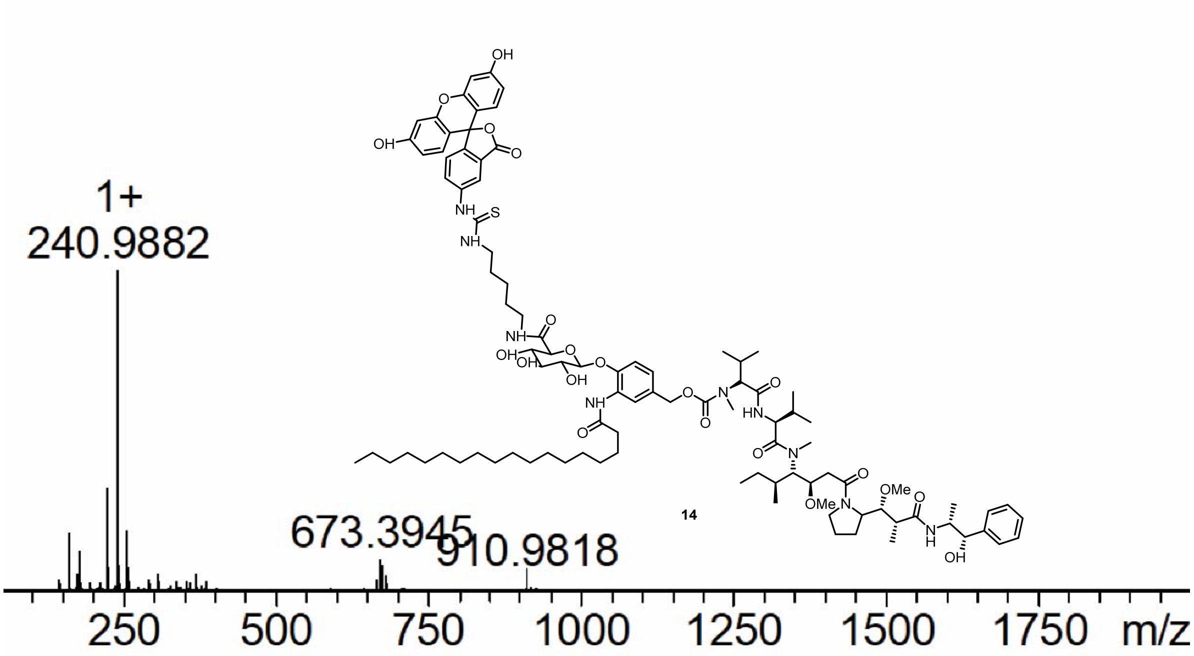
1+
240.9882
673.3945
910.9818
14
250
500
750
1000
1250
1500
1750
m/z

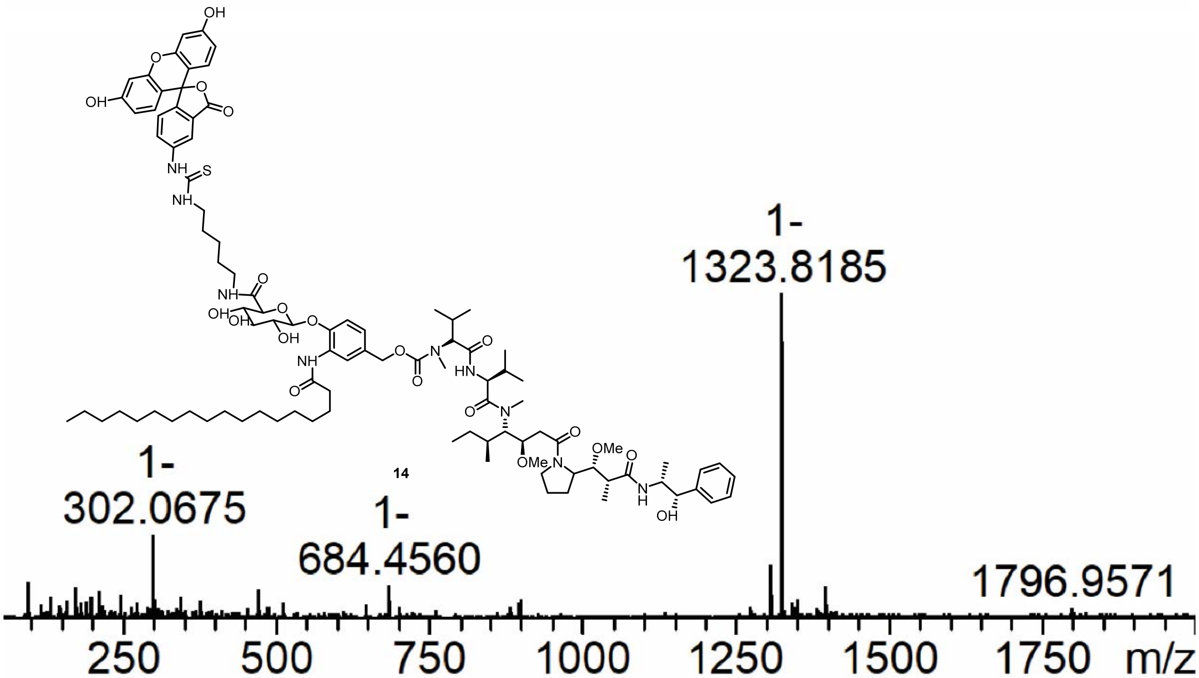
1-
1323.8185
1-
302.0675
1-
684.4560
1796.9571
14
250
500
750
1000
1250
1500
1750
m/z

## 2.5 HPLC drug release experiment

A HPLC release study was performed of Compound **11** to prove successful release of MMAE upon treatment with β-glucuronidase (GUS) enzyme. Compound **11** (0.1 mM) was dissolved in PBS (10 mM, pH = 7.4) with a 3% DMSO content. The samples were incubated at 37ºC for 2 hours either in the absence or presence of GUS enzyme (0.1 g/L). The GUS enzyme was removed by spin filtration (30K) and the samples were injected into the analytical HPLC. The HPLC run was carried out by starting at 5% eluent B. Between 0-18 minutes the eluent B content was gradually increased to 100% and eluent B was kept at 100% for 10 minutes to a total time of 28 minutes. The UV detection was performed at the wavelengths λ = 210 nm and λ = 254 nm. A reference sample with MMAE was prepared in the same manner. Results of release experiment: $t_r$ (MMAE) = 11.294 min, $t_r$ (**11** without GUS) = 21.948 min, and $t_r$ (**11** with GUS) = 11.309 min. See figure 1B.

# 3. Physics section

## 3.1 Phospholipid Vesicle and Sample Preparation

Vesicles were formed by dissolving 1:1 molar ratio of 1,2-dipalmitoyl-d62-sn-glycero-3-phosphocholine (d62-DPPC, Avanti Polar Lipids) and 1,2-dipalmitoyl-sn-glycerol-3-phospho-(1'-rac-glycerol) (DPPG, Avanti Polar Lipids) powders in solvent. Chloroform (HPLC grade, Honeywell) was used for d62-DPPC and a mixture of methanol (HPLC grade, Honeywell) and chloroform was used for DPPG. The lipids were combined in a vial briefly sonicated to mix the lipids, then dried under a steam of nitrogen and dried under a vacuum overnight. The dried lipids were resuspended in $D_2O$ (Eurisotope) and extruded 21 times through a 400 nm filter (Avanti Polar Lipids) to produce vesicles. Glu-C18-MMAE was dissolved in $D_2O$ at a concentration of 2.1 mg/mL. Samples were made for the 100 $\mu$L sample cell as follows and also can be found in **Table S1**: (1) 100 $\mu$L of vesicles in $D_2O$, (2) 50 $\mu$L of vesicles in $D_2O$ and 50 $\mu$L $D_2O$, and (3) 50 $\mu$L of vesicles in $D_2O$, 10 $\mu$L of Glu-C18-MMAE, and 40 $\mu$L $D_2O$. DLS measurements were conducted to track interaction of Glu-C18-MMAE and vesicles.

## 3.2 Dynamic light scattering (DLS)

DLS measurements were performed using a Zetasizer Nano - S90 (Malvern Instruments, UK). To 20 µL of each sample 1 mL $D_2O$ was added prior to each measurement. Each sample consisted of 3 measurements with multiple scans per measurement (determined by the software).

**Table S1**. DLS of phospholipid vesicles before and after the addition of Glu-C18-MMAE.

| Sample Name | Z-average (dia. nm) (STDEV) | PDI (STDEV) | Mean Count Rate (kcps) (STDEV) |
|---|---|---|---|
| d62-DPPC/G 100 $\mu$L | 352 (17) | 0.171 (0.013) | 227 (60) |
| d62-DPPC/G 50 $\mu$L + 10 $\mu$L R + 40 $\mu$L $D_2O$ | 327 (14) | 0.154 (0.027) | 119 (23) |
| d62-DPPC/G 50 $\mu$L + 50 $\mu$L $D_2O$ | 359 (23) | 0.223 (0.058) | 73 (32) |

Note: R = Glu-C18-MMAE

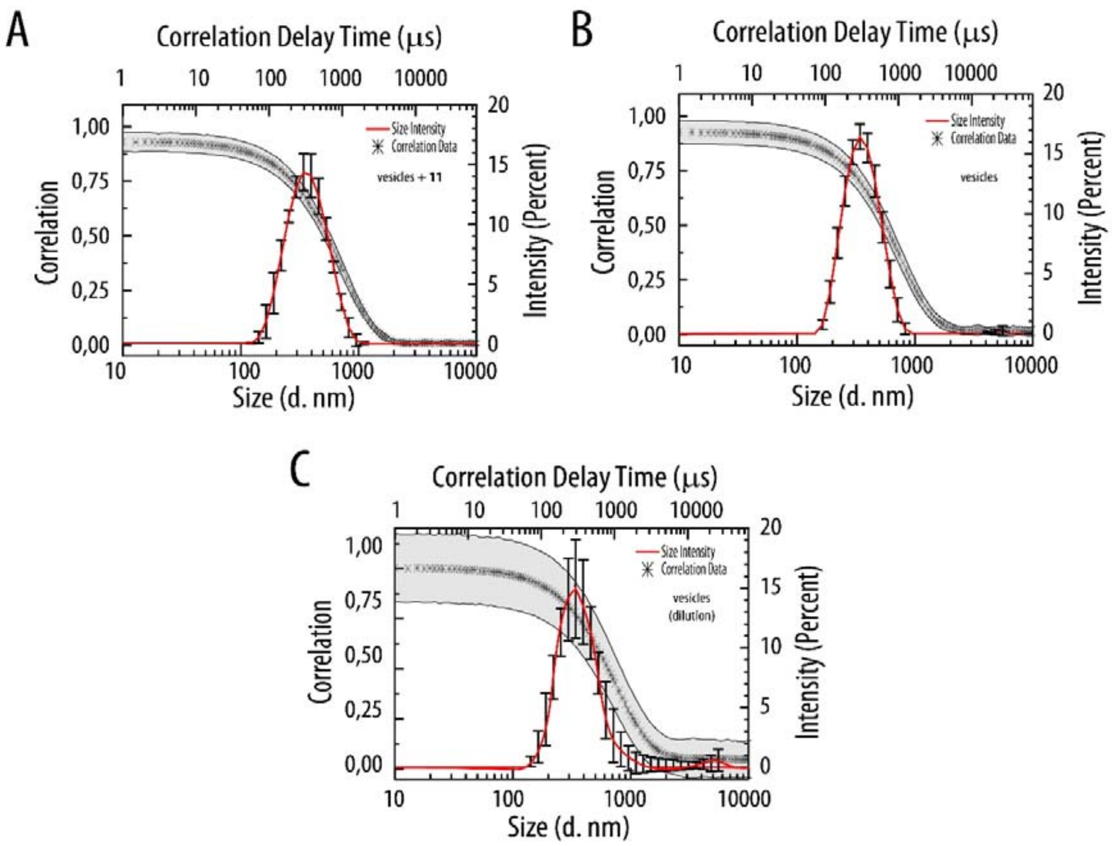


**Figure S3**. DLS data for vesicles used in SFS spectroscopy. **A)** Vesicles treated with compound 11, **B)** control vesicles, **C)** control vesicles at a dilution. Each sample consisted of 3 measurements with multiple scans per measurement (determined by the software), bars represent standard deviation.

## 3.3 Sum frequency scattering (SFS) spectroscopy

The theory describing SFS has been published in detail before.[4-6] Briefly, a broadband IR and a narrowband NIR laser pulse are overlapped in time and space to generate SF photons. Selection rules dictate that those photons are only detectable in the far field when they are generated in an ordered non-centrosymmetric medium such as interfaces. Applying this method to centrosymmetric particles in solution generate no detectable signal in the direction of the SF vector. When generating SF photons a certain fraction of the SF photons are scattered. If the detector is placed such that the detection angle is different from the SF direction, differences in path lengths of scattered photons of antipodal positions generate a phase shift that renders the inherently each other cancelling photons detectable.

The SFS setup used is based on a 7W Ti:Saph amplifier (Astrella, Coherent, USA) that generates 35fs pulses with a 1kHz repetition rate. Roughly 1.5W of the fundamental is used to pump an OPA (TOPAS prime with NDFG, Coherent, USA) generating broadband IR pulses. About 1W is used to generate the narrowband visible pulse. A pulse stretcher in 4f configuration achieves the narrowing of the broadband fundamental. A focusing mirror with 7.5cm focal length focuses both pulses to the sample. The SFS signal is collected with a collimating lens at an angle of roughly 60° off the SF direction and finally directed to the detector (Kymera 328i with iStar 334, both Andor).

Normalization was done by background subtraction and dividing by a quartz reference. Samples were then divided by mean count rate determined from DLS. The spectra were recorded in ssp (s-SF, s-VIS, p-IR) and ppp polarization combinations and each spectra was manually fit according to eq. S1[7]

$$I_{SFS}(\omega,\theta) \propto \left|\sum_n \left(A_{NR}e^{i\Delta\phi} + \frac{A_n(\theta)}{(\omega-\omega_n)+i\Upsilon_n}\right)\right|^2 \quad \text{Eq. S1}$$

Where, $A_{NR}$ is the amplitude of the weakly dispersive background, $A_n(\theta)$ the angular dependent amplitude, $\Upsilon_n$ the full width half maximum of the vibrational mode $n$, $\Delta\phi$ the phase difference between the weakly dispersive background signal and resonant signal, $\omega$ the resonant frequency, and $I_{SFS}(\omega,\theta)$ the intensity of the SFS detected photons, which are a function of resonant frequency and detection angle. The fitting parameters are recorded in Table S2.

**Table S2**. C-D and Carbonyl Region ppp and ssp Polarization SFS Fitting Results of Glu-C18-MMAE interacting with d62-DPPC/G vesicles

| Sample | $\Delta\phi$ | $A_{NR}$ | $\omega_n$ (cm$^{-1}$) | $\Upsilon_n$ (cm$^{-1}$) | $A_n$ *ppp* | $A_n$ *ssp* |
|---|---|---|---|---|---|---|
| d62-DPPC/G | 7.48 | -0.051 (ppp) 0.075 (ssp) | 2074 | 30.78 | 2.41 | 3.79 |
| | | | 2119 | 64.66 | 0.66 | 3.02 |
| | | | 2220 | 32.33 | -4.29 | 0.00 |
| | 5.21 | 0.052 (ppp) 0.048 (ssp) | 1735 | 41.74 | 6.03 | 3.33 |
| | | | | | | |
| d62-DPPC/G + GLu-C18-MMAE | 7.48 | -0.040 (ppp) 0.012 (ssp) | 2074 | 30.78 | 0.00 | 0.00 |
| | | | 2119 | 64.66 | 0.00 | 0.00 |
| | | | 2220 | 32.33 | 0.00 | 0.00 |
| | 5.21 | 0.093 (ppp) 0.005 (ssp) | 1735 | 41.74 | 2.66 | 1.32 |

## 3.4 Critical micellar concentration (CMC) of Glu-C18-MMAE

The method was adapted from Kalayanasundaram *et al.*[8]. To solutions of compound **11** in PBS was added pyrene from a stock solution of 30 mM in ethanol to a final concentration of 300 μM. Final concentration of **11** ranged from 250 to 60 μM. Fluorescence of samples were recorded between 354 and 500 nm with excitation at 334 nm in a plate reader (PerkinElmer EnSpire 2300 Multilabel Reader). Then the ratio of fluorescence at $\lambda = 383$ ($I_3$) and $\lambda = 372$ ($I_1$) was plotted as a function of concentration of **11** and used to estimate the CMC.

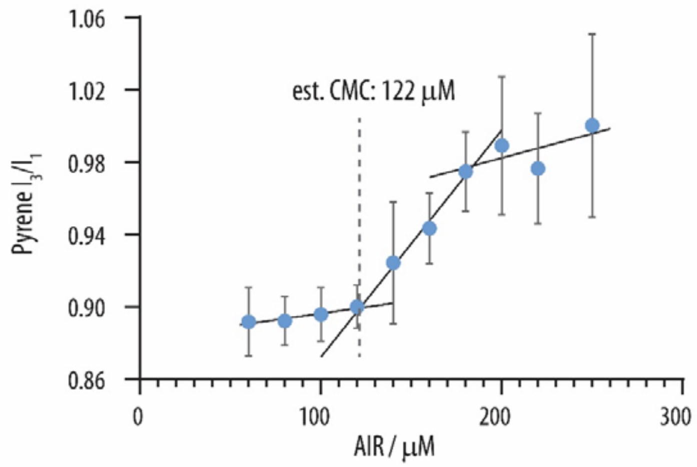


**Figure S4**. CMC determination for compound **11**. Experiment was done in triplicates (n=3), bars represent the standard deviation between measurements.

## 3.5 GUVs formation and binding studies with fluorescently labelled compounds

0.25 μg of lipids containing egg yolk phosphatidyl choline (EPC, Sigma P8318) and fluorescently labelled rhodamine (RhoL:1, Sigma 810150P) at molar ratio of 100:0.5 (EPC:RhoL) were made into a thin film on top of an ITO glass and left at vacuum overnight. Afterwards samples were hydrated with 300 μL $H_2O$ containing 300 mM sucrose. Then GUVs were formed by electroformation between ITO glasses (Nanion Technologies Vesicle Prep Pro, Freq: 10.0 Hz, Amplitude: 3.0 V, Temperature 35° C) for 3 hours. GUVs were stored at 4° C and used within 2-3 days after formation.

To label GUVs with fluorescently labelled compounds (**13** and **14**), each of the fluorescent labelling reactions was diluted in PBS 1:10. And 1 μL of this solution was added on top of 99 μL of a 1:10 dilution of GUVs in PBS (Sigma 806552). A control with GUVs which were only added solvent from the reaction mixture was also prepared. After 10 minutes, all samples were washed 3x with 300 μL of PBS by centrifugation of the GUVs (400 rcf, 45 s) and resuspended in 100 μL of PBS before directly imaging with CLSM (Zeiss LSM 700). RhoC18 lipid was observed at $\lambda_{ex}/\lambda_{em}$ 555/573 nm and fluorescein from labelled compounds at $\lambda_{ex}/\lambda_{em}$ 488/518 nm. The imaging settings were kept the same between all the samples.

# 4. Biology section

## 4.1 Cell culture

In general, cells were cultivated at 37° C in humidified air with 5% $CO_2$. After resuscitation, cells were cultured for at least 3 passages before inclusion in experiments.

## 4.2 HAP1 and bGUS knockout cell lines

Competent HAP1 cells and bGUS knockout cells were purchased form Horizon™ (HZGHC005064c00). The knockout was performed by CRISPR/Cas9 to include a 1 bp insertion in a coding exon of GUSB gene. Cells were cultured in Iscove Modified Dulbecco Media supplemented (Sigma I3390) with 10% FBS (Sigma F7524), 1% penicillin/streptomycin (Sigma P0781) and 2 mM L-glutamine (Sigma G2150). Cultures were split upon reaching 70% confluence and media was exchanged every 2-3 days as needed.

## 4.3 Spheroid formation and culture

3D cell cultures were prepared by growth of bGUS knockout cells, competent HAP1 cells, or a combination of them at a given ratio, in ultra-low attachment spheroid plates (Corning 4515). Cells were left to grow in complete media, which was exchanged every 2-3 days. 3D cultures were monitored by microscopy.

## 4.4 Determination of IC50

In a 96-well plate cells were seeded at $10^4$ cells/well and incubated for 48h. Then cells were washed with PBS and incubated with different concentrations of MMAE, Glu-MMAE and Glu-C18-MMAE (from DMSO stock solutions, DMSO concentration of all samples was kept the same at 0.8%. Control cells were added only DMSO) under three different conditions: in the presence of FBS for 48 h, in the presence of FBS for 2 h or in the absence of FBS for 30 min. The latter two conditions were washed with PBS and media was exchanged to complete media. Then samples were treated with β-glucuronidase (Sigma G8295) enzyme at 15 μg/mL. For each condition controls without the addition of enzyme were also prepared.

After 48h, viability was assessed by a metabolic assay. Briefly, PrestoBlue® reagent was diluted 1:10 in the cell media and incubated for 30 min. Then, samples were transferred to a fluorescence plate and fluorescence of resorufin was measured at $\lambda_{ex}/\lambda_{em}$ = 536/619 nm. The experiments were reproduced 3 times with at least 3 replicates each time.

## 4.4 Live/Dead staining spheroids

Spheroids were washed with PBS and incubated at room temperature with fluorescein diacetate (Sigma F7378) or propidium iodide (Sigma P4170) (4 and 6 μg/mL respectively) for 20 min. Then cells were imaged by fluorescence microscopy (Zeiss Observer.Z1). Imaging settings were set in the PI channel and adjusted on enzyme treated samples. The imaging settings were kept the same for all samples.

## 4.5 Activation of synthetic "kill switch" in 3D cultures of bGUS knockout cells

bGUS knockout at 70% confluence were washed with PBS, treated with trypsin/EDTA 0.25% (Sigma T4049) and suspended at $2x10^6$ cells/mL in serum free media. Then cells were treated with

Glu-C18-MMAE at different concentrations (0, 1, 2, 4, 8 and 16 μM) for 30 min, washed with PBS (450 g, 5 min), exchanged to complete media, seeded at $2x10^4$ cells/well in low attachment plates and 3D cultures were grown as described above. Samples were treated with β-glucuronidase enzyme at 15 μg/mL after 48h. For each sample, a control where no enzyme as added was prepared. Samples were imaged at the microscope upon live/dead staining with FDA/PI 48h after the addition of enzyme.

For time delayed activation, samples were treated with β-glucuronidase 15 μg/mL after 24, 72 and 120 hours. For each sample, a control where no enzyme as added was prepared. Samples were imaged at the microscope upon live/dead staining with FDA/PI 48h after the addition of enzyme.

## 4.6 Co-culture experiments in 3D cell cultures

bGUS knockout were treated with compound Glu-C18-MMAE at different concentrations (0, 1, 2, 4, 8 and 16 μM) as described above. Then cells were counted and mixed with HAP1 at different ratios HAP1:bGUS knockout (9:1 and 99:1) upon which $2x10^4$ cells/ well were seeded in low attachment plates and 3D cell cultures were grown.

After 48h samples were treated with glucuronidase enzyme at 15 μg/mL. For each sample, a control where no enzyme as added was prepared. Samples were imaged at the microscope upon live/dead staining with FDA/PI 48h after the addition of enzyme.

## 4.7 Confocal laser scanning microscopy of fluorescently labelled compounds on cell culture

70% confluent bGUS knockout cells were washed twice with PBS and treated with 250 μL of a 1:200 dilution of the labelling reaction mixture of compounds **13** and **14** in serum free media for 10 minutes. For untreated controls, cells were treated with a dilution from reaction mixture solvent. After incubation, cells were washed 3 times with PBS containing 10% FBS and stained with a dilution of 1:2000 in PBS of Hoescht reagent (ThermoFisher 62249) in PBS for 15 minutes at room temperature. After washing twice with PBS containing 10% FBS cells were imaged at the CLSM. Fluorescein from labelled compounds was imaged at $\lambda_{ex}/\lambda_{em}$ 488/518 nm and Hoescht at $\lambda_{ex}/\lambda_{em}$ 405/435 nm. All imaging settings were kept the same between all samples.

## 4.8 Dose response curves

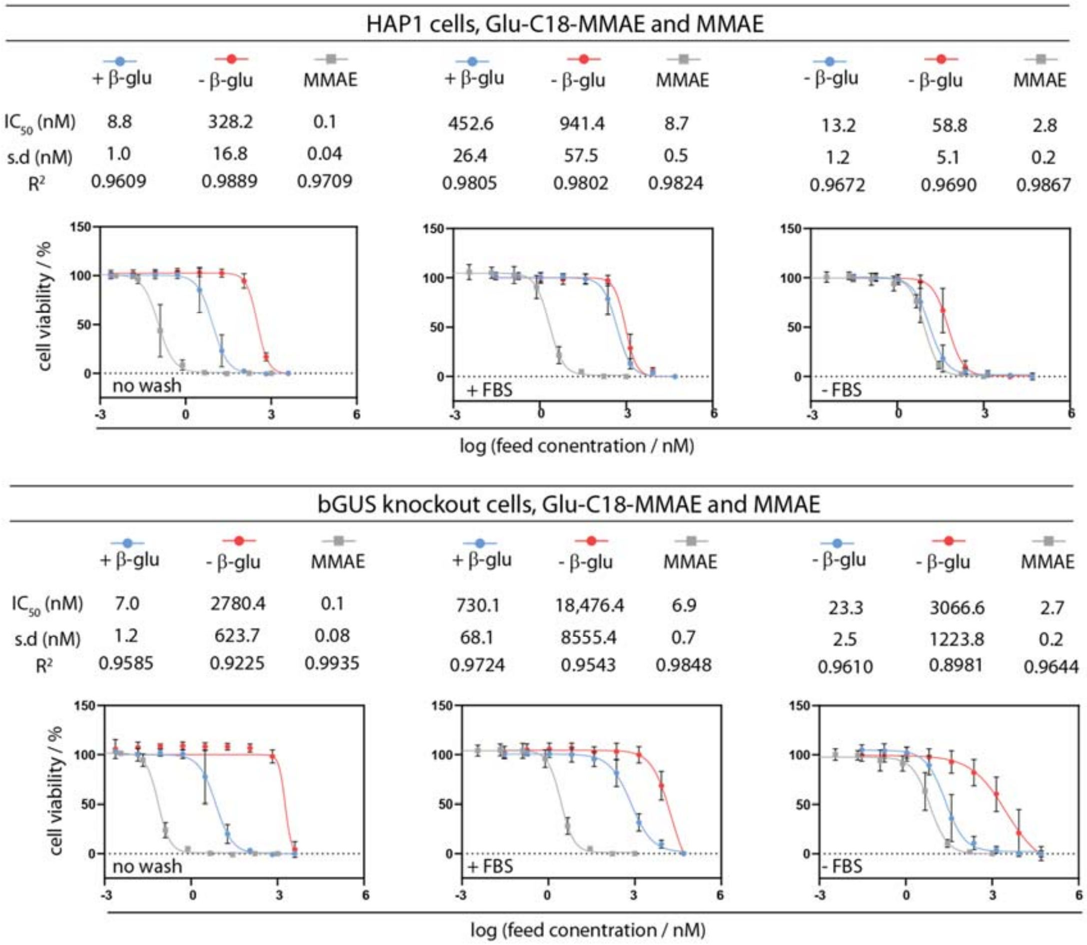


**Figure S5**. Dose response curves for Glu-C18-MMAE and MMAE in HAP1 and bGUS knockout cells (N=3, n=3 bars represent standard deviation between measurements). All curves were plotted and fitted to sigmoidal equation (variable slope, four parameters) using Graphpad Prism 8.0.1

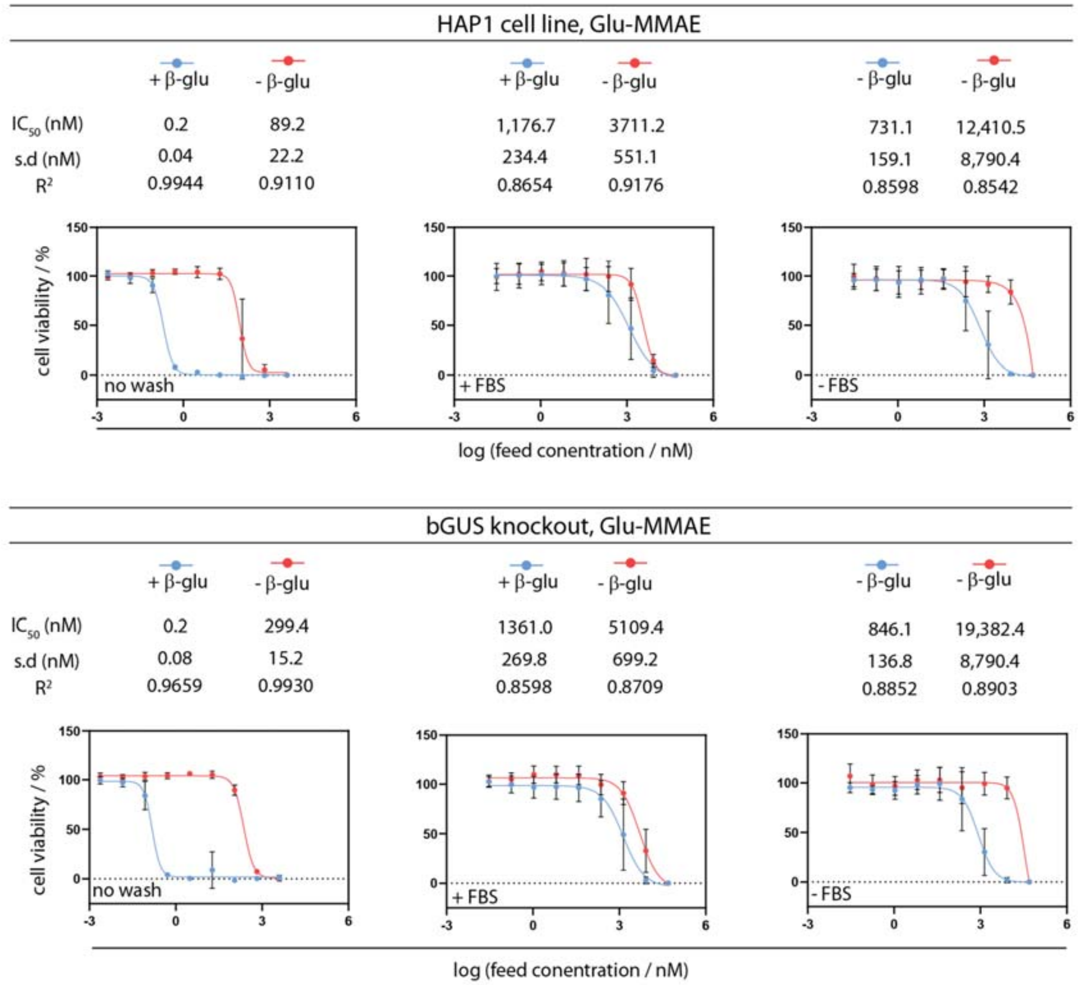


**Figure S6**. Dose response curves for Glu-MMAE in HAP1 and bGUS knockout cells (N=3, n=3, bars represent standard deviation between measurements). All curves were plotted and fitted to sigmoidal equation (variable slope, four parameters) using Graphpad Prism 8.0.1

## 4.9 Statistical analysis.

For the analysis of the dose response curves, the raw fluorescence data was treated by subtracting background and normalizing to viability controls. Then it was plotted as function of the logarithm of concentration. Then the potency was estimated by fitting to a sigmoidal curve (four parameters, variable slope) using the software Graphpad Prism 8.0.1.

For CMC determination, the three sections of the plot of pyrene $I_3/I_1$ vs concentration were fitted to straight lines with least squares using the software Graphpad Prism 8.0.1. CMC was estimated as the intersection point between the two first regions.

For DLS, each sample consisted of 3 measurements with multiple scans per measurement (determined by the software), bars represent standard deviation. Normalization of SFS data was done by background subtraction and dividing by a quartz reference. Samples were then divided by mean count

rate determined from DLS. The spectra are fit with equation S1 using the equation stated in the SFS section and using software IgorPro 6.37.

# 5. References

1. Jarlstad Olesen, M. T.; Walther, R.; Poier, P. P.; Dagnæs-Hansen, F.; Zelikin, A. N., Molecular, Macromolecular, and Supramolecular Glucuronide Prodrugs: Lead Identified for Anticancer Prodrug Monotherapy. *Angewandte Chemie International Edition n/a* (n/a).

2. Walther, R.; Nielsen, S. M.; Christiansen, R.; Meyer, R. L.; Zelikin, A. N., Combatting implant-associated biofilms through localized drug synthesis. *J Control Release* **2018,** *287*, 94-102.

3. Walther, R.; Jarlstad Olesen, M. T.; Zelikin, A. N., Extended scaffold glucuronides: en route to the universal synthesis of O-aryl glucuronide prodrugs. *Org Biomol Chem* **2019,** *17* (29), 6970-6974.

4 Roke, S.; M. Bonn; A. V. Petukhov, Nonlinear optical scattering: The concept of effective susceptibility. *Physical Review B* **2004,** *70* (11), 115106.

5.Roke, S.; W. G. Roeterdink; J. E. G. J. Wijnhoven; A. V. Petukhov; A. W. Kleyn; M. Bonn, Vibrational sum frequency scattering from a submicron suspension. *Physical review letters* **2003,** *91* (25), 258302.

6. de Beer, A. G. F.; S. Roke; J. I. Dadap, Theory of optical second-harmonic and sum-frequency scattering from arbitrarily shaped particles. *JOSA B* **2011,** *28* (6), 1374-1384.

7.Strader, M. L.; H. B. De Aguiar; A. G. F. de Beer; S. Roke, Label-free spectroscopic detection of vesicles in water using vibrational sum frequency scattering. *Soft Matter* **2011,** *7* (10), 4959-4963.

8. K. Kalyanasundaram, J. K. Thomas, Environmental Effects on vibronic band intensitites in yrene monomer fluorescence and their application in studies of micellar systems. *J. Am. Chem. Soc.* 1977, 99, 2039-2045.
